# A Novel Hierarchical Clustering Approach for Joint Analysis of Multiple Phenotypes Uncovers Obesity Variants Based on ARIC

**DOI:** 10.3389/fgene.2022.791920

**Published:** 2022-03-22

**Authors:** Liwan Fu, Yuquan Wang, Tingting Li, Siqian Yang, Yue-Qing Hu

**Affiliations:** ^1^ Center for Non-communicable Disease Management, National Center for Children’s Health, Beijing Children’s Hospital, Capital Medical University, Beijing, China; ^2^ State Key Laboratory of Genetic Engineering, Human Phenome Institute, Institute of Biostatistics, School of Life Sciences, Fudan University, Shanghai, China; ^3^ Shanghai Center for Mathematical Sciences, Fudan University, Shanghai, China

**Keywords:** GWAS, hierarchical clustering, multiple phenotypes, obesity, bioinformatics

## Abstract

Genome-wide association studies (GWASs) have successfully discovered numerous variants underlying various diseases. Generally, one-phenotype one-variant association study in GWASs is not efficient in identifying variants with weak effects, indicating that more signals have not been identified yet. Nowadays, jointly analyzing multiple phenotypes has been recognized as an important approach to elevate the statistical power for identifying weak genetic variants on complex diseases, shedding new light on potential biological mechanisms. Therefore, hierarchical clustering based on different methods for calculating correlation coefficients (HCDC) is developed to synchronously analyze multiple phenotypes in association studies. There are two steps involved in HCDC. First, a clustering approach based on the similarity matrix between two groups of phenotypes is applied to choose a representative phenotype in each cluster. Then, we use existing methods to estimate the genetic associations with the representative phenotypes rather than the individual phenotypes in every cluster. A variety of simulations are conducted to demonstrate the capacity of HCDC for boosting power. As a consequence, existing methods embedding HCDC are either more powerful or comparable with those of without embedding HCDC in most scenarios. Additionally, the application of obesity-related phenotypes from Atherosclerosis Risk in Communities *via* existing methods with HCDC uncovered several associated variants. Among these, *UQCC1*-rs1570004 is reported as a significant obesity signal for the first time, whose differential expression in subcutaneous fat, visceral fat, and muscle tissue is worthy of further functional studies.

## Introduction

The applications of genome-wide association studies (GWASs) have successfully established a large number of genetic variants associated with numerous complex diseases (Lutz et al., 2017), contributing to the understanding of the mechanisms of complex diseases such as obesity (Locke et al., 2015; Shungin et al., 2015). Notably, GWASs usually apply the univariate analysis to examine the association between genetic variants and a single phenotype, and in general, multiple phenotypes related to diseases are typically collected together for better understanding the physiological process of diseases (Yang et al., 2010). For example, information about individual status of obesity, insulin resistance, hypertension, and atherosclerotic dyslipidemia is required jointly to explore metabolic syndrome (Sattar et al., 2008). A research of hypertension inevitably takes account of the magnitude of systolic blood pressure (SBP) and diastolic blood pressure (DBP) (Yang and Wang, 2012), From the aspect of pleiotropy, namely, some genes could simultaneously affect multiple related phenotypes, the significance of biological process emphasizes the importance of multiple phenotypes analyses. Univariate analysis means conducting single phenotype separately and showing the outcomes for each phenotype (O'Reilly et al., 2012). However, analyzing one phenotype at each time will absolutely suffer multiple testing corrections, which results in a power loss in GWASs (Yang et al., 2010). Recently, jointly analyzing multiple phenotypes together has become popular due to its increased statistical power of identifying genetic variants compared to analyzing each phenotype separately, enhancing the magnitude of explanation for the biological progress of relevant diseases, and elevating the credibility of the results (Yang et al., 2010; Aschard et al., 2014; Fu et al., 2021).

In the past decade, joint analysis of multiple phenotypes has developed rapidly, which may roughly be classified into three categories: regression approaches, integrating testing statistics from univariate analyses, and variable reduction approaches (Yang and Wang, 2012). Tests that fall into the first category, regression approaches, mainly encompass three different methods to analyze the association of multiple phenotypes with a genetic variant: mixed effect models (Bates and DebRoy, 2004), frailty models (Therneau et al., 2003), and generalized estimating equations (Zeger and Liang, 1986). In the second category, integrating testing statistics from univariate analyses, as the name suggests, integrates different test statistics or *p*-values from univariate association analyses via various strategies (Schaid et al., 2016; Yang et al., 2016). Nowadays, various approaches of integrating test statistics or *p*-values from univariate analyses have been established to investigate the association between genetic variants and multiple phenotypes concerning the correlation structure among phenotypes (van der Sluis et al., 2013; Kwak and Pan, 2016; Liang et al., 2016; Yang et al., 2016). In the last category, tests on the basis of variable reduction approaches roughly adopt three dimension reduction techniques. The first one is the principal component analysis (PCA) (Aschard et al., 2014). In PCA, the first few principal components (PCs) with regard to majority of the total phenotype variance are selected for evaluating their association with a genetic variant. The second one is the canonical correlation analysis (CCA) (Tang and Ferreira, 2012). CCA supplies an efficient and powerful method for both univariate and multivariate analyses ignoring the need for permutation test in association studies by searching for linear combinations that maximize the association between two classes of multidimensional variables. The last one is the principal component of heritability (PCH) (Ott and Rabinowitz, 1999; Klei et al., 2008; Wang et al., 2016). PCH adopts a linear combination of phenotypes that represents the highest heritability among all linear combinations of phenotypes for reducing multiple phenotypes.

In this study, we develop a novel variable reduction approach called hierarchical clustering based on different methods for calculating correlation coefficients (HCDC) aiming at jointly analyzing multiple phenotypes. By means of a dimension reduction technique, HCDC constructs a typical phenotype from each cluster of phenotypes, then applies the existing approaches for jointly analyzing multiple phenotypes to estimate the genetic associations with the typical phenotypes instead of the individual phenotypes. The vital significance in dimension reduction technique of HCDC is that when one cluster is composed of positively highly correlated phenotypes, every linear combination of phenotypes is a representative of the cluster reasonably (Bien and Wegkamp, 2013; Bühlmann et al., 2013). One specific advantage of HCDC is that it does not need to know individual phenotypes, and it actually requires a similarity matrix about the phenotypes. In real data analysis, the similarity matrix of phenotypes can be evaluated from the summary statistical values with regard to the usage of independent single nucleotide polymorphisms (SNPs) in a GWAS (Zhu et al., 2015). Previously, hierarchical clustering method (HCM) is also a clustering approach (Liang et al., 2018). However, when calculating the correlation coefficients between distinct clusters, HCM adopts the uniform expression of correlation coefficients, not concerning the number of phenotypes in each cluster. As a result, HCM obtains lower statistical power in some scenarios. On the contrary, we propose HCDC by virtual extensive simulations to reveal the validity of the improved two-step approach and to explore its power. Notably, the performance of three existing approaches employing HCDC or HCM, namely, multivariate analysis of variance (MANOVA) (Cole and MaxwellScott, 1994), joint model of multiple phenotypes (MultiPhen) (O'Reilly et al., 2012), trait-based association test that uses extended Simes procedure (TATES) (van der Sluis et al., 2013), is compared with that of without employing HCDC or HCM. In this way, scientific issues about whether there exists an advantage of clustering (MANOVA, MultiPhen, and TATES using HCDC or HCM are compared with these approaches without using HCDC or HCM) and which clustering approach has more obviously outstanding performance (MANOVA, MultiPhen, and TATES using HCDC are compared with these approaches using HCM) can be solved. Our simulations reveal that MANOVA, MultiPhen, and TATES employing HCDC have correct type Ⅰ error rates and possess more power than MANOVA, MultiPhen, and TATES without employing HCDC in most simulation scenarios. Finally, we emphatically explore the performance of HCDC approach by utilizing the obesity-related phenotypes from a real dataset, Atherosclerosis Risk in Communities (ARIC) Study (Author Anonymous, 1989) from dbGaP. Consequently, a total of eight significant SNPs are detected, and subsequent bioinformatics analysis is carried out for better understanding the results. From another point of view, the interesting results indicate the effective performance of HCDC in real data application.

## Methods

### Proposed HCDC

Assume a sample with *N* individuals, and *M* phenotypes $Y_{1},Y_{2},\ldots ,Y_{M}$ . Meanwhile, let $X={\left(x_{1},\ldots ,x_{N}\right)}^{\text{T}}$ denote the genotypic score of *N* individuals at a genetic variant of interest, where $x_{i}\in \text{\{0, }1\text{, }2\text{\}}$ represents the number of minor alleles that *i* th subject carries at that variant.

Note that the key issue in the hierarchical clustering is to specify a measure of similarity between disjoint groups of phenotypes. Now let us take two disjoint clusters $G_{1}$ and $G_{2}$ of phenotypes as an example to demonstrate the calculation of similarity between these two groups. Denote $M_{1}$ and $M_{2}$ as the numbers of phenotypes in $G_{1}$ and $G_{2}$ , respectively.

1. If $M_{1}=M_{2}=1$ , Pearson correlation coefficient (Jin and Lin, 2019) between two phenotypes is calculated to represent the similarity between $G_{1}$ and $G_{2}$ .
2. If $M_{1}=1$ and $M_{2}>1$ , or $M_{1}>1$ and $M_{2}=1$ multiple correlation coefficient (Cohen and Cohen, 1983; Jin and Lin, 2019) is employed based on the phenotypes involved in $G_{1}$ and $G_{2}$ , respectively, to reveal the similarity between a pair of clusters.
3. If $M_{1}>1$ and $M_{2}>1$ , canonical correlation coefficient (Ferreira and Purcell, 2009) is applied according to the phenotypes involved in $G_{1}$ and $G_{2}$ respectively to show the similarity between two clusters.

Once we have the similarity measure between two clusters of phenotypes, we apply a hierarchical clustering approach to cluster the phenotypes. Specifically, following the agglomerative (bottom–up) procedure, we start at the bottom (i.e., the lowest level) where each phenotype is a cluster and then recursively merge a selected pair of clusters with the biggest intergroup similarity at the next lower level into a single cluster. This produces a grouping at the next higher level with one less cluster until all phenotypes are grouped as one cluster at the highest level. Finally, there are *M* − 1 levels in the hierarchy.

For any *b*, 1≤b≤M−1 , let $h_{b}$ denote the height at the level *b* in the dendrogram, which is the biggest intergroup similarity at the level *b* − 1. Similar to a proposed principle (Bühlmann et al., 2013), a stopping criterion is adopted to determine the optimal number *K* of clusters,

$$K=\arg\underset{1\leq b\leq M-2}{\min}\left(h_{b+1}-h_{b}\right).$$

Without loss of generality, the corresponding *K* clusters are denoted as $G_{1},G_{2},\ldots ,G_{K}$ .

The established HCDC encompasses the following two steps. First, *M* phenotypes are grouped into *K* clusters as aforementioned, and each of the *K* clusters singles out a representative phenotype. Second, existing approaches to the *K* representative phenotypes instead of the original *M* phenotypes are employed to evaluate the genetic association of multiple phenotypes with a genetic variant.

Notice that each phenotype should be scaled first before constructing the representative phenotype for each other. We define the representative phenotype for the *k*th cluster as the mean phenotype values in the cluster, namely

$${\bar{Y}}^{\left(k\right)}=\frac{1}{M_{k}}\sum_{m\in G_{k}}Y_{m},k=1,\ldots ,K,$$

where $M_{k}$ is the number of phenotypes in the cluster $G_{k},k=1,2,\ldots ,K$ . Denote $\bar{Y}$ as the N×K design matrix whose *k*th column is given by ${\bar{Y}}^{\left(k\right)}$ . Then, existing approaches are employed to evaluate the association between $\bar{Y}$ and *X*.

The source code for HCDC approach can be found in https://github.com/YQHuFD/HCDC.

### Comparison of Methods

For convenience, let $1_{n}$ denote the ones vector of length *n* and $0_{n}$ represent the all zeroes vector of length *n*, where *n* is a positive integer. First, we need to introduce one of the potential competitors, HCM (Liang et al., 2018). Same as the process of HCDC, HCM also adopts the bottom–up hierarchical clustering method on the basis of the similarity. But unlike HCDC, HCM defines the similarity matrix with $S_{ij}$ , where $S_{ij}$ is the *i j*th entry of the sample correlation matrix of *M* phenotypes $Y_{1},Y_{2},\ldots ,Y_{M}$ . The average linkage is employed as the similarity between two clusters in HCM. To be precise, the similarity between clusters $G_{k}$ and $G_{l}$ (which are two disjoint subsets of {1, 2, … , *M*}) is given by

$$\frac{1}{M_{k}\cdot M_{l}}\sum_{i\in G_{k},j\in G_{l}}S_{ij},$$

where $M_{k}$ and $M_{l}$ are the numbers of phenotypes in the respective clusters $G_{k}$ and $G_{l}$ , 1≤k,l≤K .

Except the different definition of similarity between pairs of clusters, the remaining processes of HCM are exactly the same as the HCDC. Second, the performance of MANOVA (Cole and MaxwellScott, 1994), MultiPhen (O'Reilly et al., 2012), and TATES (van der Sluis et al., 2013) with using HCDC is compared with that of with using HCM and that of without using HCDC/HCM approaches. The ones with employing HCDC and HCM are referred as HCDCMANOVA, HCMANOVA, HCDCMultiPhen, HCMultiPhen, HCDCTATES, and HCTATES, respectively. In the following, we briefly review the existing approaches for easy reference.

MANOVA (multivariate analysis of variance) (Cole and MaxwellScott, 1994): A total of *M* phenotypes are involved in the standard MANOVA and the background variance–covariance matrix Σ including M×M symmetrical elements is unconstrained. There are ((M+1)×M)/2 freely evaluated elements in the covariances and variances. Standard MANOVA tests the null hypothesis that the *M* regression coefficients are all zeroes, which asymptotically follows *F* distribution.

MutiPhen (joint model of multiple phenotypes) (O'Reilly et al., 2012): In the MultiPhen model, the genotypes and phenotypes are treated as ordinal response and predictors, respectively. Likelihood ratio test is performed to test the null hypothesis in the proportional odds logistic regression.

TATES (trait-based association test that uses extended Simes procedure) (van der Sluis et al., 2013): The *p*-values from univariate analysis is integrated to get a comprehensive *p*-value, and simultaneously, correlation between phenotypes is considered for adjustment. Denote $\min(M_{e}p_{\left(j\right)}/M_{e(j)})$ as the *p*-value of TATES, where $p_{\left(j\right)}$ represents the $j^{th}\left(j=1,\ldots ,M\right)$ ascending sorted *p*-value; $M_{e}$ and $M_{e\left(j\right)}$ are the effective number of independent *p*-values among all involved *M* phenotypes and *j* specific phenotypes, respectively. The correlation matrix of *p*-values is derived to obtain the effective numbers.

## Results

### Simulation Studies

Suppose that a population is in Hardy–Weinberg equilibrium (HWE), and we generate the genotypes of the genetic variants following the binomial distribution with parameter two and the minor allele frequency (MAF). This simulation study sets MAF = 0.3 in most scenarios. We generate multiple phenotypes by means of the following factor model (van der Sluis et al., 2013):

$$y=\lambda x+dc\gamma f+d\sqrt{1-c^{2}}\times \varepsilon ,$$

where $y={\left(y_{1},\ldots ,y_{M}\right)}^{T}$ denotes the *M* phenotypes; *x* is the genotype; $\lambda ={\left(\lambda_{1},\ldots ,\lambda_{M}\right)}^{T}$ represents the vector of values suggesting the effects of genetic variant on the *M* phenotypes; *f* shows the vector of factors; $f={\left(f_{1},\ldots ,f_{R}\right)}^{T}∼MVN\left(0,\Sigma \right)$ , $\Sigma =\left(1-\rho \right)I+\rho 1_{R}1_{R}^{T}$ ; *I* is the identity matrix; *R* represents the number of factors, and ρ is the correlation between factors; γ is an M×R matrix; *d* is a diagonal matrix for correcting the variance of phenotypes; *c* denotes a constant; $\varepsilon ={\left(\varepsilon_{1},\ldots ,\varepsilon_{M}\right)}^{T}$ represents a vector of random errors, and $\varepsilon_{1},\ldots ,\varepsilon_{M}$ are mutually independent and follow the standard normal distributions. Consider the following four models with different numbers of factors affected by genotypes.

Model 1: There is only one factor, and the genotype has an effect on all phenotypes with the same effect size. That is, R=1 , $\lambda =\beta 1_{M}$ , $d=diag\left(1_{M}\right)$ , and $\gamma =1_{M}$ .
Model 2: There are two factors and the genotype impacts on one factor with the same effect. Namely, R=2 , $\lambda ={\left(0_{M/2}^{T},\beta 1_{M/2}^{T}\right)}^{T}$ , $d=diag\left(1_{M}\right)$ , and $\gamma =bdiag\left(1_{M/2},1_{M/2}\right)$ , which is the block diagonal matrix of $1_{M/2}$ and $1_{M/2}$ .
Model 3: There are four factors, and the genotype has an effect on the last two factors with varied effect directions. That is, R=4 , $\lambda ={\left(0_{M/2}^{T},-\beta 1_{3M/16}^{T},\beta 1_{M/4}^{T},-\beta 1_{M/16}^{T}\right)}^{T}$ ,

$$\gamma =bdiag\left({\left(1_{3M/16}^{T},-1_{M/16}^{T}\right)}^{T},{\left(1_{3M/16}^{T},-1_{M/16}^{T}\right)}^{T},{\left(1_{3M/16}^{T},-1_{M/16}^{T}\right)}^{T},{\left(1_{3M/16}^{T},-1_{M/16}^{T}\right)}^{T}\right),$$

and

$$d=diag\left({\left(\frac{8}{M}{\left[1:M/4\right]}^{T},\frac{8}{M}{\left[1:M/4\right]}^{T},\frac{8}{M}{\left[1:M/4\right]}^{T},\frac{8}{M}{\left[1:M/4\right]}^{T}\right)}^{T}\right)$$

where [1:M/4] denotes the vectors of components 1,2,…,M/4 .

Model 4: There are four factors, and the genotype has an influence on the last three factors with different sizes. Namely, R=4 ,

$$\lambda ={\left(0_{M/4}^{T},\frac{2\beta }{M/4+1}{\left[1:M/4\right]}^{T},-\beta 1_{3M/16}^{T},\beta 1_{M/4}^{T},-\beta 1_{M/16}^{T}\right)}^{T}$$

$$\gamma =bdiag\left({\left(1_{3M/16}^{T},-1_{M/16}^{T}\right)}^{T},1_{M/4}^{T},{\left(1_{3M/16}^{T},-1_{M/16}^{T}\right)}^{T},{\left(1_{3M/16}^{T},-1_{M/16}^{T}\right)}^{T}\right),$$

and

$$d=diag\left({\left(\frac{8}{M}{\left[1:M/4\right]}^{T},\frac{8}{M}{\left[1:M/4\right]}^{T}\text{ ,}\frac{8}{M}{\left[1:M/4\right]}^{T}\text{ ,}\frac{8}{M}{\left[1:M/4\right]}^{T}\right)}^{T}\right)$$

For the all models, the within-factor correlation is $c^{2}$ , and the between-factor correlation is $\rho c^{2}$ . For evaluating type Ⅰ error rates and powers, this study sets *N* = 2,000 unrelated individuals, and the number of phenotypes *M* = 16, 32. According to β=0 , all phenotypes independent of genotypes are generated to estimate the type Ⅰ error rates of all investigated approaches, encompassing MANOVA, MultiPhen, TATES, HCMANOVA, HCMultiPhen, HCTATES, HCDCMANOVA, HCDCMultiPhen, and HCDCTATES. The corresponding Q–Q plots of type Ⅰ error rates in varied approaches are shown in Supplementary Figures S1–8. Notably, for assessing powers, we do not only alter the values of β (meanwhile, the within-factor correlation $c^{2}=0.5$ and between-factor correlation $\rho c^{2}=0.1$ ) but also vary the values of within-factor correlation $c^{2}=0.3,0.5,0.7$ , and 0.9 (meanwhile, the between-factor correlation $\rho c^{2}=0.1$ ).

### Simulation Results

We establish varied nominal significance levels, distinct number of phenotypes, and different number of factors to assess the type Ⅰ error rates of all the nine methods. In each simulation model, the *p*-values of all these evaluated methods are estimated by their asymptotic distributions. The type Ⅰ error rates of MANOVA, MultiPhen, TATES, HCMANOVA, HCMultiPhen, HCTATES, HCDCMANOVA, HCDCMultiPhen, and HCDCTATES are evaluated by 10,000 replicated samples. For 10,000 replicated samples, we calculate that the 95% confidence intervals (CIs) for type Ⅰ error rates in the nominal levels of 0.01 and 0.05 are about (0.008, 0.012) and (0.0457, 0.0543), respectively. The estimated type Ⅰ error rates of all these tested methods are shown in Table 1 (*M* = 16) and Table 2 (*M* = 32). We observe that the majority of the type Ⅰ error rates of HCDCMANOVA, HCDCMultiPhen, and HCDCTATES are within 95%CIs, which reflects the validity of the established HCDC applied to existing methods. Additionally, the type Ⅰ error rates of MANOVA, MultiPhen, TATES, HCMANOVA, HCMultiPhen, and HCTATES are not obviously deviated from the nominal levels. For more information, please see the Q–Q plots in Supplementary Figures S1–8.

**TABLE 1:** Evaluations of type Ⅰ error rates of the nine methods in four simulation models.

Type Ⅰ error rates								
Methods	Model 1		Model 2		Model 3		Model 4	
	*α* = 0.01	*α* = 0.05	*α* = 0.01	*α* = 0.05	*α* = 0.01	*α* = 0.05	*α* = 0.01	*α* = 0.05
HCDCMANOVA	0.0102	0.0523	0.0113	0.0522	0.0086	0.0532	0.0095	0.05
HCMANOVA	0.01	0.0517	0.0113	0.0524	0.0094	0.0478	0.0101	0.0509
MANOVA	0.0108	0.0505	0.0112	**0.0547**	0.0089	0.0514	0.0103	0.0519
HCDCMultiPhen	0.0101	0.0538	0.012	0.0527	0.0089	0.0532	0.0102	0.0483
HCMultiPhen	0.0091	0.0528	**0.0121**	0.0526	0.0102	0.0519	0.0101	0.0494
MultiPhen	0.0107	0.0523	0.0116	0.052	0.0094	0.0517	0.011	0.0537
HCDCTATES	0.0108	0.0502	0.0112	0.0511	0.0099	0.0466	0.0112	0.0506
HCTATES	**0.0122**	0.051	0.0114	0.0512	0.0109	0.0488	0.0103	0.05
TATES	0.0111	0.0473	0.0119	0.0512	0.0112	0.0514	0.0121	0.0535

Sample size *N* = 2,000, the number of phenotypes *M* = 16, *c* ^2^ = 0.5, *ρc* ^2^ = 0.1, and minor allele frequency (MAF) = 0.3. The type Ⅰ error rates of all nine methods are evaluated by 10,000 replicated samples at the significance of *α*. The values in bold indicate that the type Ⅰ error rates are out of 95% CI of the nominal significance level.

**TABLE 2:** Evaluations of type Ⅰ error rates of the nine methods in four simulation models.

Type Ⅰ error rates								
Methods	Model 1		Model 2		Model 3		Model 4	
	*α* = 0.01	*α* = 0.05	*α* = 0.01	*α* = 0.05	*α* = 0.01	*α* = 0.05	*α* = 0.01	*α* = 0.05
HCDCMANOVA	0.01	0.0515	0.0118	0.0543	0.01	0.0498	0.0099	0.048
HCMANOVA	0.0111	0.0502	0.0118	**0.0544**	0.0111	0.0503	0.0102	0.0506
MANOVA	0.0101	0.051	0.0106	**0.0582**	0.0115	**0.0545**	0.0102	0.0515
HCDCMultiPhen	0.0099	0.0502	0.0117	**0.0545**	0.0098	0.05	0.0091	0.0497
HCMultiPhen	0.011	0.0516	0.0119	0.0543	0.0102	0.0503	0.0099	0.0512
MultiPhen	0.0102	0.0495	0.011	**0.0589**	0.0115	**0.0573**	0.0106	0.0511
HCDCTATES	0.0112	0.0514	0.0119	0.0539	0.0097	0.0483	0.0086	0.0463
HCTATES	0.0093	**0.045**	0.012	0.0538	0.0111	**0.0546**	0.0106	0.0516
TATES	**0.0078**	**0.041**	0.0105	0.0465	**0.0128**	0.0524	0.0101	0.0496

Sample size *N* = 2,000, the number of phenotypes *M* = 32, *c* ^2^ = 0.5, *ρc* ^2^ = 0.1, and minor allele frequency (MAF) = 0.3. The type Ⅰ error rates of all nine methods are evaluated by 10,000 replicated samples at the significance of *α*. The values in bold indicate that the type Ⅰ error rates are out of 95% CI of the nominal significance level.

**FIGURE 1:**
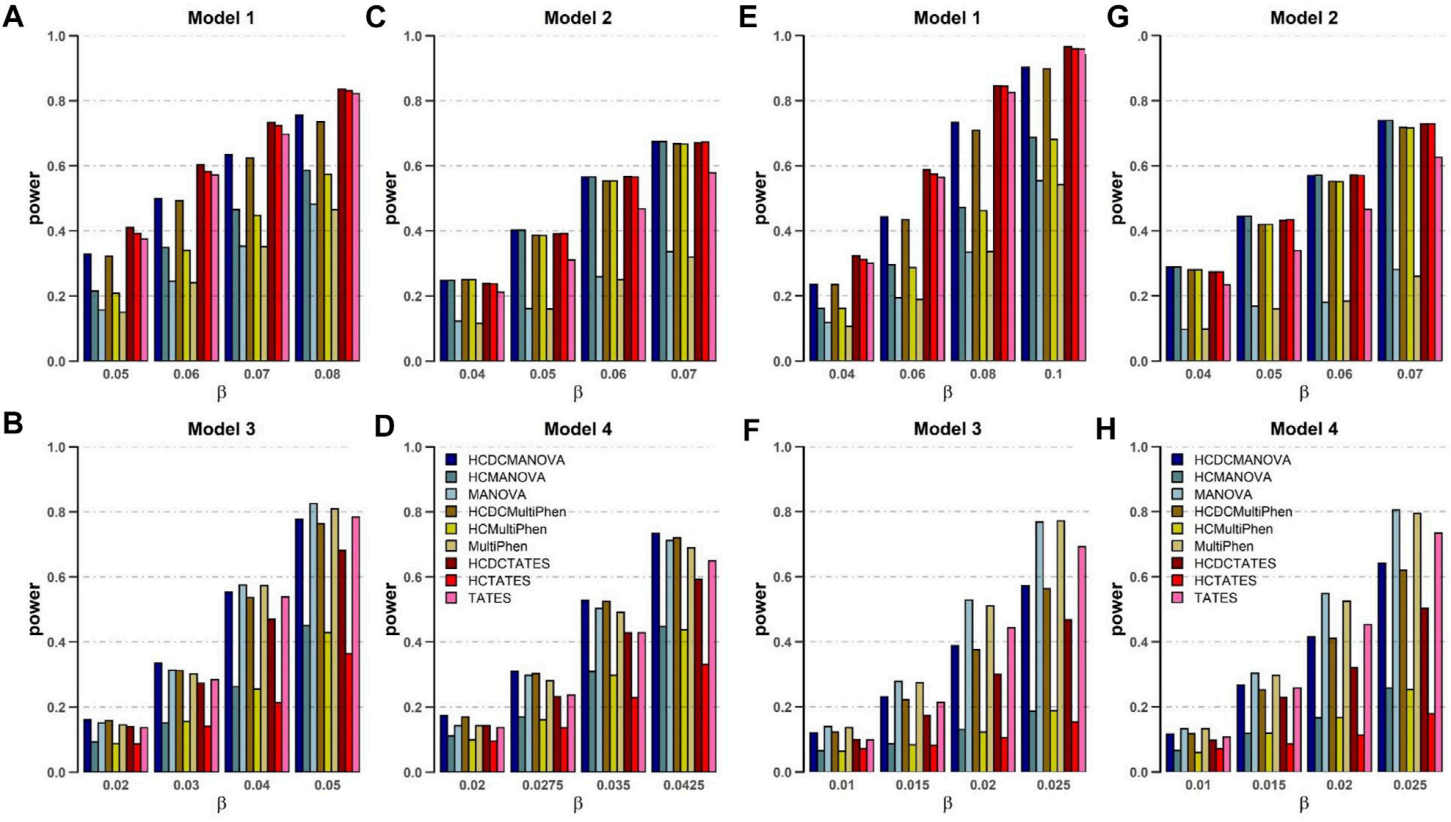
Power comparisons of the nine methods as a function of β in the four models. Sample size *N* = 2,000, the number of phenotypes *M* = 16 **(A–D)** and M = 32 **(E–H)**, c^2^ = 0.5, ρc^2^ = 0.1, and MAF = 0.3. The power of all the methods is evaluated by 1,000 replicated samples at a significance level of 0.05.

For power comparison for these nine methods, we alter distinct numbers of phenotypes and different models. The powers of all tests are estimated on the basis of 1,000 replications and 2,000 subjects at a significance level of 0.05. From the plots of power against genetic effect *β* (Figure 1), the following are observed and can be shown:

1. When the genetic variant has the same effect on all the phenotypes (Model 1), HCDCMANOVA, HCDCMultiPhen, and HCDCTATES are powerful than HCMANOVA, HCMultiPhen, and HCTATES, respectively. Meanwhile, HCMANOVA, HCMultiPhen, and HCTATES are powerful than MANOVA, MultiPhen, and TATES, respectively. In most replications, HCDC and HCM cluster various phenotypes into one or several categories to reduce the number of phenotypes to be analyzed for enhancing the power of test. Obviously, HCDC is slightly powerful than HCM in this scenario.

2. When the genetic effects on phenotypes reveal some groups and possess the same direction (Model 2), the power of HCDCMANOVA, HCDCMultiPhen, and HCDCTATES is equal to that of HCMANOVA, HCMultiPhen, and HCTATES, respectively. However, MANOVA, MultiPhen, and TATES with HCDC or HCM are much more powerful than MANOVA, MultiPhen, and TATES, respectively. These results indicate that clustering can definitely increase the power of test.

3. When the genetic effects on phenotypes appear in some groups and show different directions (Models 3 and 4), MANOVA, MultiPhen, and TATES are powerful than MANOVA, MultiPhen, and TATES with HCDC or HCM, respectively.

4. No matter altering of genetic effects *β* or changes in correlation coefficients between varied phenotypes, HCDCMANOVA and HCDCMultiPhen, HCMANOVA and HCMultiPhen, MANOVA and MutiPhen have similar performance in all four models, respectively.

5. When the genetic effects on phenotypes show obvious same direction within a group (Models 1 and 2), HCDCTATES, HCTATES, and TATES have better performance than other approaches.

From the within-factor correlation *c* ^2^ (Supplementary Figures 9, 10), we can observe the following:

6. When the genetic variant has the same effect on the phenotypes within a group, and there exists the same variance among phenotypes within this group, the powers of all estimated methods decrease as the within-factor *c* ^2^ increases (Models 1 and 2). However, our proposed MANOVA, MultiPhen, and TATES with using HCDC have obvious advantage over MANOVA, MultiPhen, and TATES without using HCDC, respectively.

7. When the genetic variant has the distinct effects on the phenotypes within a group, and there are different variances among phenotypes within this group (Models 3 and 4), MANOVA, MultiPhen, and TATES with using HCDC have more power than MANOVA, MultiPhen, and TATES without using HCDC as the within-factor *c* ^2^ is <0.5, but MANOVA and MultiPhen get more advantage as *c* ^2^ is >0.5, which reveal that MANOVA and MultiPhen take heteroscedasticity between different phenotypes into account when calculating genetic associations.

In summary, the existing approaches employing HCDC has controlled type Ⅰ error rates and have more advantage over or are comparable with those without employing HCDC. Therefore, we could draw that our established HCDC could give more power than HCM or original approaches without using HCDC, and in some scenarios, the advantage is more obvious. In other scenarios, the existing methods using HCDC is comparable with the most powerful test.

### Real Data Analysis

We use our established approach, HCDC, together with other existing methods to the real data analysis in ARIC study (Author Anonymous, 1989). Briefly, ARIC is a prospective cohort study supported by the National Heart, Lung, and Blood Institute (NHLBI), aiming at assessing atherosclerosis risk in community. It keeps track of the altering of the occurrence of atherosclerosis-relevant diseases and cardiovascular risk factors in different regions, races, genders, and periods of time, in order to explore the natural process of atherosclerosis (Morrison et al., 2013). We acquire the clinical phenotypic and genotyped data of ARIC from dbGaP server of the United States National Center for Biotechnology Information (accession number: phs000090.v4.p1).

To evaluate the performance of HCDC together with other existing methods in analyzing real data, we evaluate the nine approaches to explore obesity-related phenotypes in ARIC. We choose nine continuous phenotypes concerning obesity comprising body weight, body mass index (BMI), mean skinfold thickness of the triceps brachii, average subscapular skinfold thickness, hip girth, waist, waist-to-hip ratio (WHR), calf girth, and wrist breadth and three covariates of age, gender, and race. The description of these variables is shown in Table 3 in detail, and the correlation matrix of obesity-related phenotypes is displayed in Supplementary Figure S11. A total of 12,701 individuals across 272,027 SNPs are left to be analyzed subsequently after removing subjects with missing data under any of these 12 variables together with the genetic variants concerning missing rate more than 0.2 or HWE <10^–4^. Each phenotype is adjusted for those three covariates by conducting the linear regression model.

**TABLE 3:** The descriptions of involved obesity-related phenotypes and covariates in ARIC.

Index	All	Gender			Race		
		Male	Female	*p* Value	White	Black	*p* value
*N*	12771	5,704	7067	—	9,633	3,138	—
Male, %	44.66	—	—	—	47.02	37.44	**9.11 × 10** ^**–21**^
Age, years	54.09 ± 5.73	54.450 ± 5.75	53.76 ± 5.69	**6.76 × 10** ^**–13**^	54.34 ± 5.68	53.34 ± 5.80	**5.51 × 10** ^**–17**^
Weight, lb	173.13 ± 36.85	188.27 ± 31.46	160.92 ± 36.36	**<2.2 × 10** ^**–16**^	169.61 ± 35.69	183.99 ± 38.25	**1.90 × 10** ^**–74**^
Weight missing, %	0.149	0.158	0.142	0.995	0.083	0.351	**0.002**
BMI, kg/m^2^	27.66 ± 5.30	27.54 ± 4.18	27.75 ± 6.05	**0.020**	27.01 ± 4.86	29.65 ± 6.05	**9.98 × 10** ^**–104**^
BMI missing, %	0.149	0.158	0.142	0.995	0.083	0.351	**0.002**
Triceps, mm	25.26 ± 10.02	19.34 ± 7.87	30.04 ± 8.97	**<2.2 × 10** ^**–16**^	24.54 ± 9.08	27.48 ± 12.23	**1.73 × 10** ^**–34**^
Triceps missing, %	0.157	0.175	0.142	0.798	0.093	0.351	**0.004**
Scapular, mm	24.48 ± 11.59	22.22 ± 9.19	26.31 ± 12.92	**1.13 × 10** ^**–94**^	21.85 ± 9.33	32.59 ± 13.89	**1.60 × 10** ^**–299**^
Scapular missing, %	0.446	0.561	0.354	0.107	0.353	0.733	**0.009**
WC, cm	96.94 ± 13.83	99.23 ± 10.93	95.09 ± 15.54	**1.25 × 10** ^**–68**^	96.19 ± 13.33	99.25 ± 15.02	**5.34 × 10** ^**–24**^
WC missing, %	0.141	0.123	0.156	0.798	0.104	0.255	0.092
HC, cm	104.55 ± 10.31	102.85 ± 8.09	105.93 ± 11.63	**2.81 × 10** ^**–68**^	103.50 ± 9.478	107.79 ± 11.98	**7.52 × 10** ^**–72**^
HC missing, %	0.141	0.140	0.142	0.999	0.104	0.255	0.092
WHtR	0.926 ± 0.078	0.963 ± 0.054	0.895 ± 0.081	**<2.2 × 10** ^**–16**^	0.928 ± 0.079	0.920 ± 0.076	**4.66 × 10** ^**–8**^
WHtR missing, %	0.149	0.140	0.156	0.999	0.114	0.255	0.131
Calf, cm	37.44 ± 3.67	38.06 ± 3.17	36.95 ± 3.95	**1.48 × 10** ^**–68**^	37.39 ± 3.58	37.60 ± 3.93	**0.006**
Calf missing, %	0.157	0.210	0.113	0.248	0.114	0.287	0.062
Wrist, mm	53.62 ± 5.18	57.78 ± 3.66	50.27 ± 3.53	**<2.2 × 10** ^**–16**^	53.59 ± 5.26	53.74 ± 4.91	0.137
Wrist missing, %	0.117	0.123	0.113	0.999	0.073	0.255	**0.022**

*N* is the number of subjects; BMI, is body mass index; Triceps is average skinfold thickness of triceps brachii; Scapular is mean subscapular skinfold thickness; WC, is waist; HC, is hip girth; WHtR is waist-to-hip ratio; Calf is calf girth; and Wrist is wrist breadth. The distributions of normal index are described by mean ± standard deviation; the distributions of non-normal indicators are described by means (25% quantile, 75% quantile). For normal distribution indicators, the differences between groups are estimated using the *t*-test (the variances of two groups are homogeneous) or the approximate *t*-test (the variances of two groups are heterogeneous). For non-normally indicators, Wilcoxon signed-rank test is used to test the differences between indicators to get the *p*-values of differences. For discrete indicators, the chi-square test is used for hypothesis testing and then deriving *p*-values. Bold number indicates *p* < 0.05. ARIC, atherosclerosis risk in communities.

**FIGURE 2:**
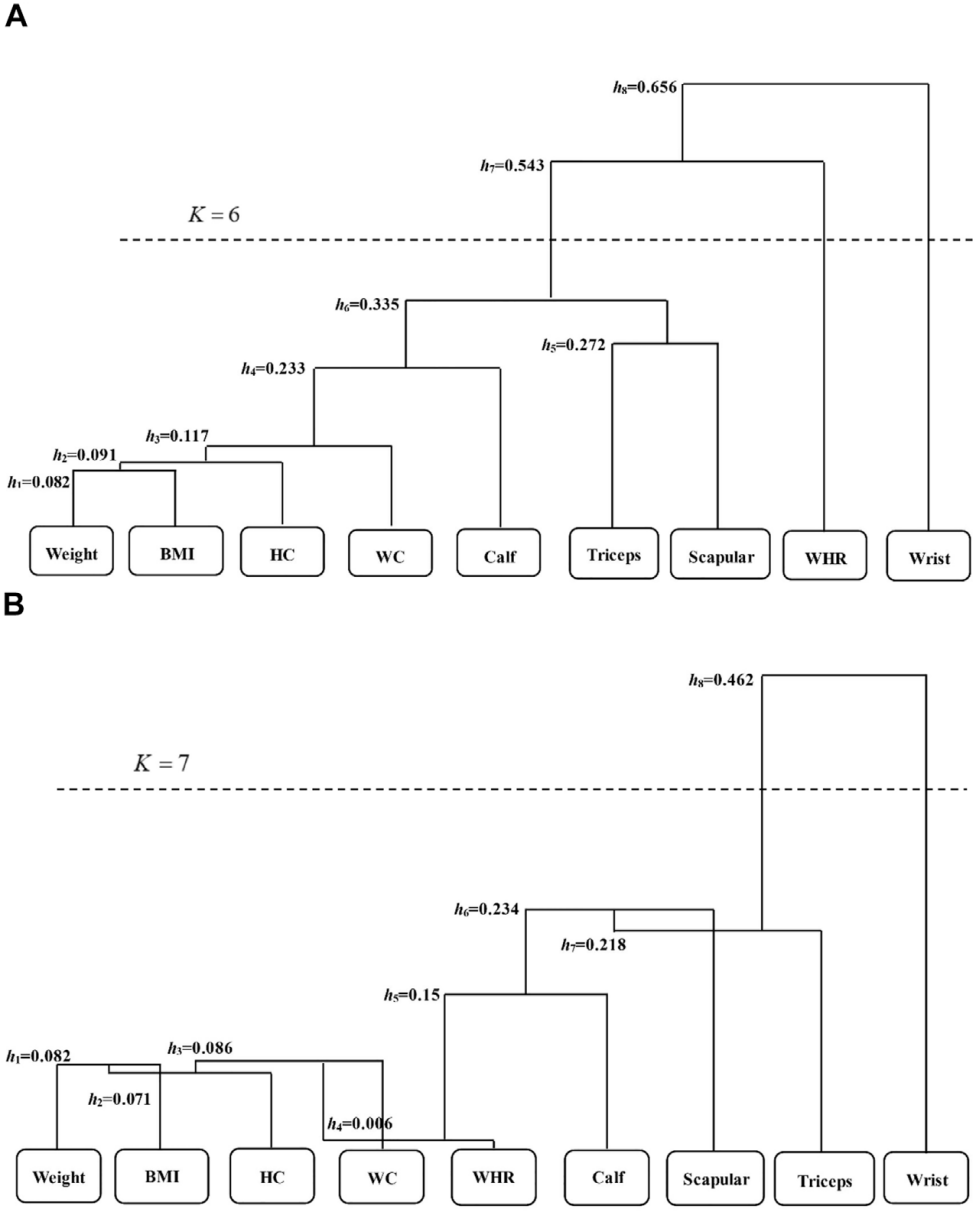
The dendrogram of the nine phenotypes in the ARIC study *via* HCM **(A)** and HCDC **(B)**. h represents the maximum value of correlation coefficient in each clustering process, which is taken as the “branch length” of the clustering tree. K reveals the final clustering times according to the stopping criteria. BMI is body mass index; Triceps is average skinfold thickness of triceps brachii; Scapular is mean subscapular skinfold thickness; WC is waist; HC is hip girth; WHR is waist-to-hip ratio; Calf is calf girth; and Wrist is wrist breadth.

According to the scaled phenotypes with respect to obesity, we use these nine methods to identify associated genetic variants. Due to multiple testing correction, we apply the genome-wide significance threshold of 5 × 10^–8^. HCDC clusters nine phenotypes into two groups in this real data analysis, one only containing wrist breadth, while the other includes the rest. As comparisons, three groups are obtained after clustering by HCM, one only containing wrist breadth, and another encompasses WHR phenotype, while the other contains the remaining phenotypes. The dendrogram of clustering process for HCM and HCDC in ARIC data are presented in Figure 2. From these graphs, we can observe that there are significant differences between the HCM method and the HCDC method we proposed in the clustering process. Specifically, when the correlation coefficients between different clusters are calculated, the correlation coefficients increase with the increase in clustering times in HCM (*h* is gradually increasing), while in HCDC, the correlation coefficients may increase, or they may decrease compared to the last clustering result. These differences can be explained by the distinct ways to calculate the correlation coefficients. HCM uses a uniform formula to evaluate the similarity between pairs of clusters. However, pairs of clusters generally include varied situations, comprising single phenotype versus single phenotype, single phenotype versus multiple phenotypes, or multiple phenotypes versus multiple phenotypes. Nevertheless, HCDC takes those into account fully to deal with complex and changeable situations; as a result, such clustering result may be more convincing for most of circumstances.

A total of eight SNPs are identified as significant signals for at least one method (Table 4). Previously, a large amount of studies (Frayling et al., 2007; Heard-Costa et al., 2009; Lindgren et al., 2009; Meyre et al., 2009; Thorleifsson et al., 2009; Willer et al., 2009; Heid et al., 2010; Speliotes et al., 2010; Bradfield et al., 2012; Wen et al., 2012; Berndt et al., 2013; Monda et al., 2013; Locke et al., 2015; Shungin et al., 2015) have covered that *FTO* contributes to the risk of obesity due to the population-based studies and the relevant experiments elaborating specific mechanisms. Among the eight associated SNPs, rs9939609 and rs8050136 are located in *FTO* gene. In addition, *UQCC* region is covered to be associated with height (Sanna et al., 2008). Few other SNPs have been explored to assess the association with obesity or obesity-related phenotypes. From Table 4, we can observe that HCDCMANOVA identified three SNPs; HCMANOVA identified two SNPs; MANOVA identified four SNPs; HCDCMultiPhen identified three SNPs, more than the number of SNPs identified by HCMultiPhen (twoSNPs) and MultiPhen (one SNP); HCDCTATES identified three SNPs; TATES identified four SNPs; while no SNP was identified by HCTATES. Overall, the results in real data analysis are highly consistent with the simulation performance. The number of SNPs identified by existing methods with HCDC is comparable with the largest number of SNPs identified by existing methods without HCDC. In order to make the overall performance clearer in real data results, we draw Q–Q plots and Manhattan plots after the application of these nine different methods in ARIC data (Supplementary Figures S12–18). From these plots, we can intuitively observe the SNPs identified by distinct methods, and their *p*-values in the same plot to compare their sizes.

**TABLE 4:** Display of significant SNPs and the corresponding *p*-values in the analysis of ARIC.

Chr	SNP	HCDCMANOVA	HCMANOVA	MANOVA	HCDCMultiphe	HCMultiphen	Multiphen	HCDCTATES	HCTATES	TATES
3	rs17017947	0.873	0.184	**1.02 × 10** ^**–11**^	NA	NA	NA	0.803	0.690	0.314
10	rs41470552	0.102	0.004	**6.25 × 10** ^**–9**^	NA	NA	NA	0.285	0.748	0.0358
11	rs7927943	1.72 × 10^–7^	1.88 × 10^–7^	5.57 × 10^–6^	1.88 × 10^–7^	1.21 × 10^–7^	3.33 × 10^–6^	9.18 × 10^–8^	0.513	**1.16 × 10** ^**–8**^
11	rs1945647	5.83 × 10^–7^	2.49 × 10^–7^	1.19 × 10^–5^	4.26 × 10^–7^	1.12 × 10^–7^	6.27 × 10^–6^	2.31 × 10^–7^	0.554	**1.77 × 10** ^**–8**^
16	rs9939609	**1.67 × 10** ^**–11**^	**9.53 × 10** ^**–11**^	**1.85 × 10** ^**–8**^	**2.98 × 10** ^**–11**^	**1.67 × 10** ^**–10**^	**3.39 × 10** ^**–8**^	**1.68 × 10** ^**–11**^	0.331	**2.97 × 10** ^**–10**^
16	rs8050136	**3.83 × 10** ^**–11**^	**2.10 × 10** ^**–10**^	**4.29 × 10** ^**–8**^	**8.07 × 10** ^**–11**^	**4.33 × 10** ^**–10**^	8.66 × 10^–8^	**1.11 × 10** ^**–10**^	0.277	**2.86 × 10** ^**–9**^
20	rs201561	**1.06 × 10** ^**–8**^	5.18 × 10^–8^	2.48 × 10^–6^	**1.11 × 10** ^**–8**^	5.45 × 10^–8^	2.91 × 10^–6^	2.57 × 10^–7^	0.861	7.99 × 10^–7^
20	rs1570004	1.07 × 10^–7^	4.86 × 10^–7^	5.28 × 10^–5^	1.54 × 10^–7^	7.06 × 10^–7^	7.77 × 10^–5^	**1.97 × 10** ^**–8**^	0.864	6.12 × 10^–8^

The *p*-values of nine methods are calculated based on asymptotic distribution. *p*-Value <5 × 10^–8^ are in bold. “NA” reveals MultiPhen is not available because the genotype at the specified SNP does not take all three values of 0, 1, and 2 in these data. SNP, single-nucleotide polymorphism; ARIC, atherosclerosis risk in communities.

### Characteristics of the Significant Variants

We searched the annotations of the associated SNPs on the basis of the Ensemble website (https://asia.ensembl.org) and SCAN website (http://scandb.org), which are shown in Table 5. From Table 5, it can be observed that these significant SNPs are located in intergenic or intron region, and some of them have been covered to be associated with BMI, type 2 diabetes, or height. In general, the first or large-scale GWASs have reported some of these associated signals. The ID of PubMed could be inquired to retrieve the relevant progress of these SNPs. Additionally, there is no influence for us to explore the expressions of the genes that the significant SNPs are associated with, although most of them are located in the intron or intergenic region. Moreover, most of these significant SNPs reveal that their possible effects on the expressions of corresponding genes based on the cell lines of HapMap CEU and YRI (Table 5).

**TABLE 5:** The annotations of the significant identified SNPs.

SNPs	Chr	Position (GRCh38)	Alleles (alt/Ref)	Gene (nearest)	Feature	Expression genes	Reported (yes/No)	Reported phenotypes	GWAS references
rs17017947	3	276171	A/C	*CHL1*	Intron	—	No	—	—
rs41470552	10	102222133	T/G	*PITX3*	Intergenic	—	No	—	—
rs1945647	11	81602715	C/T	*MTND6P25*	Intergenic	*GNAI2,STK40,LIMK1,LIG4, HLTF,ZNF511,CBLL1,NUDT17, POLR3C,DAGLB,KDELR2,NUP93, PRCC,C16orf80,RAB33B,LRP8*	No	—	—
rs7927943	11	81637194	C/T	*MTND6P25*	Intergenic	*WSCD2,GNAI2,ZFHX3,NUP93, FAM60A,LIMK1,MAP4, FLJ31958,LIG4,HLTF*	No	—	—
rs8050136	16	53782363	C/A	*FTO*	Intron	*HES7,LATS2*	Yes	BMI, T2D, Adiposity	PMID:18372903
									PMID:31217584
									PMID:19079260
rs9939609	16	53786615	T/A	*FTO*	Intron	*CR1,CR1L,ZNRF1,ANKRD50, LATS2,TSPYL4,HES7*	Yes	BMI, T2D	PMID:17434869
									PMID:31217584
									PMID:17554300
rs1570004	20	35370450	A/T	*UQCC*	Intron	—	Yes	Height	PMID:18193045
rs201561	20	22018575	G/C	*RPL41P1*	Intergenic	*P2RX3,EHD4*	Yes	Balding type 1	PMID:30595370

Annotations are from Ensemble website (https://asia.ensembl.org) and SCAN website (http://scandb.org); intron denotes the SNP is located between exons; intergenic denotes the SNP is located between genes. Expression genes denote annotations added after analysis of transcriptional levels of eQTL in cell lines from HapMap CEU and YRI samples using Affymetrix human exon 1.0 ST array; GWAS references indicate the identifications of PubMed. SNP, single-nucleotide polymorphism; GWAS, genome-wide association study; eQTL, expression quantitative trait locus.

**FIGURE 3:**
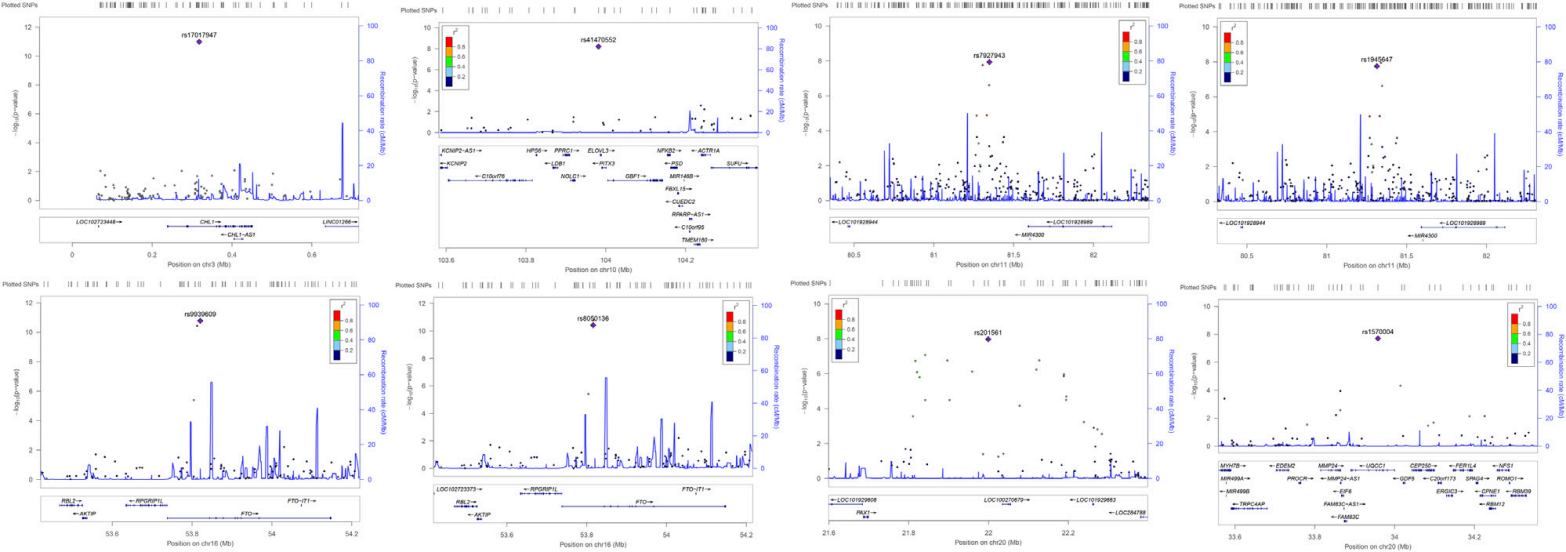
The regional association plots of the significant SNPs identified in ARIC. The *p*-values of rs17017947 and rs41470552 are evaluated by MANOVA method. The *p*-values of 7927943 and rs1945647 are estimated by TATES method. The *p*-values of rs9939609, rs8050136, and rs201561 are assessed by HCDCMANOVA method. The *p*-values of rs1570004 is evaluated by HCDCTATES. LD is constructed using the hg19 version of the 1000 Genome (American). The plots where rs7927943 and rs1945647 are located show the 1,000-kb range around these most significant SNPs. The plots where the rest SNPs (rs7927943, rs1945647, rs9939609, rs8050136, rs201561, and rs1570004) are located present the 400-kb range around these identified significantly SNPs. SNP, single-nucleotide polymorphism.

For more extensive investigation of whether the significant SNPs identified in ARIC have LD with the other nearby loci, that is, to detect the correlations between these eight associated significantly SNPs in this study with the undetectable surrounding loci, we produced regional plots presented in Figure 3. From Figure 3, it is clear that rs7927943 is physically close to rs1945647, and their LD is quite robust, which reflects that their *r* ^2^ is >0.8. What is more, both of them are located near the *LOC101928989* gene, regulating the expressions of certain genes (*LIMK1*, *GNAI2*, etc.). Since both rs7927943 and rs1945647 manipulate corresponding expressions of genes, subsequently, the relationship between these SNPs and obesity can be studied from the perspective of gene expression. Notice that both SNPs rs9939609 and rs8050136 are located in *FTO* gene attaching to chromosome 16, and their physical regions are close to each other with a high correlation *r* ^2^ >0.8 (Figure 3). It is well known that rs9939609 acts as an obese variant (Frayling et al., 2007). Because of the strong LD between rs9939609 and rs8050136, it is reasonable to speculate that rs8050136 is also associated with obesity-related phenotypes. Three SNPs, namely, rs17017947, rs1570004, and rs41470552, are located in the intron region of genes *CHL1*, *UQCC*, and *NOLC1*, respectively. None of them possesses relatively strong LD with the surrounding loci, so these SNPs probably have an effect on corresponding phenotypic characteristics independently. The rs201561 around *LOC100270679* has a profound LD with the surrounding loci (Figure 3), combined with the fact that the association result of *p*-value for rs201561 is the smallest among all the nearby variants, revealing that the surrounding loci have an impact on the phenotypes because of the high LD with rs201561.

**FIGURE 4:**
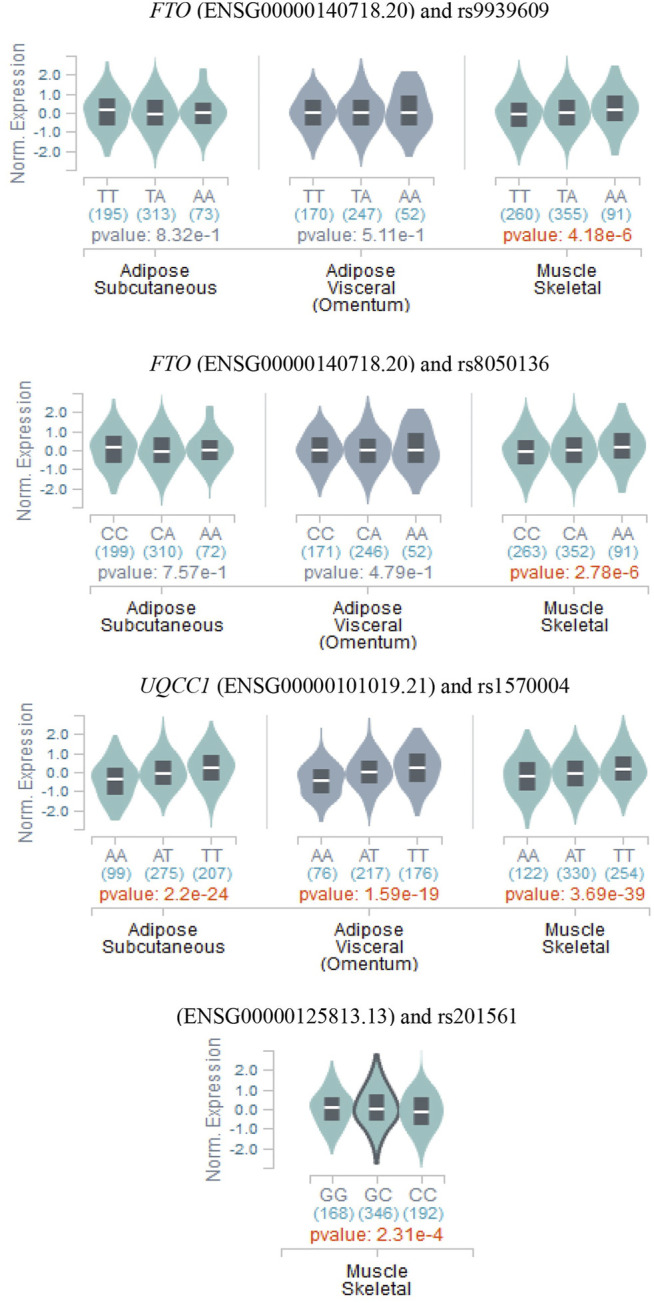
The relationship between the genotypes of the significant SNPs discovered by HCDC method and eQTL in subcutaneous adipose tissue, visceral adipose tissue, and muscle tissue (data are from GTEx website).

With the purpose of exploring the SNPs associated with obesity-related phenotypes, and the expressions of those identified by all the methods employed in this study in different adipose tissues, we retrieved the relevant content of GTEx website (https://www.gtexportal.org/home/). Consequently, the significant SNPs (rs17017947, rs41470552, rs7927943, and rs1945647) not identified by existing methods with HCDC have not been detected to be expressed in relevant tissues *via* GTEx query, while these distinct genotypes of significant SNPs (rs9939609, rs8050136, rs201561, and rs1570004) identified by existing methods with HCDC present differential expressions in adipose tissue or muscle tissue (Figure 4). In other words, the proposed HCDC has certain significance for biological research from the perspective of gene expression. Furthermore, it is noteworthy that the different genotypes of *UQCC1*-rs1570004 are differentially expressed in subcutaneous adipose, visceral adipose, or muscle tissue (*p* < 1.59 × 10^–19^). Moreover, the phenotypes adopted in real data analysis denote various measurement phenotypes about obesity, so the differentially expressed tissues are highly consistent with the phenotypes adopted in this study. Thus, *UQCC1*-rs1570004, as a SNP that has not been reported to be associated with obesity-related phenotypes in other studies so far, is worthy of further functional experimental studies in the future to confirm its impressive value.

## Discussion

In this article, HCDC is proposed to jointly analyze multiple phenotypes in association analyses. The established approach employs the similarity measure to cluster multiple phenotypes. Using HCDC, we apply the existing methods to detect the genetic associations with the combined phenotypes rather than the individual phenotypes. HCDC owns several obvious advantages compared to other dimension reduction approaches. First, a dendrogram involved in the multiple phenotypes can be produced by HCDC (see Figure 2), which could supply more information about the structure of phenotypes. Second, not limited to the correlation coefficients, any proper measurements of distance can be used for the hierarchical clustering procedure, although the specific effects are worth further consideration. Third, HCDC is computationally fast, so it is easy to implement. Notably, HCDC does not need to acquire the individual phenotypes, and on the contrary, it only acquires the similarity matrix of phenotypes. This similarity of matrix can be evaluated from the test statistics of summary data employing the independent SNPs in a GWAS (Zhu et al., 2015). This is a major advantage of HCDC clustering using correlation coefficients between phenotypes.

We performed extensive simulations together with the real data analysis to assess the performance of MANOVA, MultiPhen, and TATES combined with applying HCDC and compared these with their original versions. The simulation results reveal that these three methods applying HCDC not only possess correct type Ⅰ error rates but also own more advantage over these without applying HCDC under a series of simulation scenarios. For more realistic simulation settings, GCTA software is the first choice. Thus, further tests should be evaluated in the future (Yang et al., 2011). More importantly, the real data analysis results elucidate that HCDC shows great potential in multiple phenotypes analysis of ARIC *via* GWAS about obesity, and the bioinformatics analysis for these results also supports them. In addition, we also use another clustering method, HCM, as a major competitor to compare its performance with that of HCDC. We suggest that the most important thing for HCM to be improved is that when calculating the correlation coefficient between two clusters, it should take the imbalanced numbers of phenotypes in two clusters into account, and it may not be appropriate to use a unified calculation formula of correlation coefficient. In real data analysis, the fact that the performance of HCDC is better than HCM confirms our point of view. Presently, HCDC is more suitable for continuous phenotypes. After the transformation of phenotypes, it can also be applied to dichotomous or mixed traits. However, its performance in dichotomous or mixed traits situation still needs to be further investigated.

Then, we use HCDC to analyze ARIC data and discovered that *UQCC1*-rs1570004 has a significant correlation with multiple phenotypes about obesity traits. Bioinformatics exploration shows that varied genotypes of *UQCC1*-rs1570004 are differentially expressed in subcutaneous fat, visceral fat, and muscle tissue (*p* < 1.59 × 10^–19^). The differentially expressed tissues are consistent with the phenotypes studied in this work. Therefore, *UQCC1*-rs1570004, as an SNP that has not been reported to be associated to obesity-related phenotypes in the literature, is worthy of further functional experiments in the future to confirm its potential value. From the perspective of application in real data, HCDC owns certain value and significance for further association studies.

In summary, HCDC is an effective approach for the association study between multiple phenotypes and genetic variants in varied research fields. In medical research, many research disciplines have strong intersection. Generally, different disciplines carry out the association study between phenotypes and genetic variants separately. Interdisciplinary research on multiple phenotypes, such as phenotypes across multiple tissues, including various indicators with behavior, morphology, and physiology, will be likely extended to phenome research (Houle et al., 2010), which would be very meaningful. Because there is no assumption for HCDC in the aspect of genetic effect model, clustering multiple phenotypes into different categories according to similarity measure between phenotypes in HCDC is very useful for phenome research. Moreover, in a large number of phenotypes, HCDC does not need to understand the specific model for generating data, while only understanding the correlation matrix between phenotypes is undoubtedly another decent feature. In reality, it is common that the genetic structure among different phenotypes is complex and usually unknown. HCDC provides an effective and novel research strategy for exploring high-dimensional phenotypic data in the coming era of phenome as shown in simulations.

## Data Availability

Publicly available datasets were analyzed in this study. These data can be found here: The datasets ARIC for this study can be found in the dbGaP https://www.ncbi.nlm.nih.gov/projects/gap/cgi-bin/study.cgi?study id = phs000090.v4.p1.
