# Supplementary material for: A Novel Hierarchical Clustering Approach for Joint Analysis of Multiple Phenotypes Uncovers Obesity Variants Based on ARIC
Source: Front Genet. 2022 Mar 22;13:791920. doi: 10.3389/fgene.2022.791920 (PMC8981031; doi:10.3389/fgene.2022.791920)
Supplement: Supplementary file 1 [file DataSheet1.PDF]

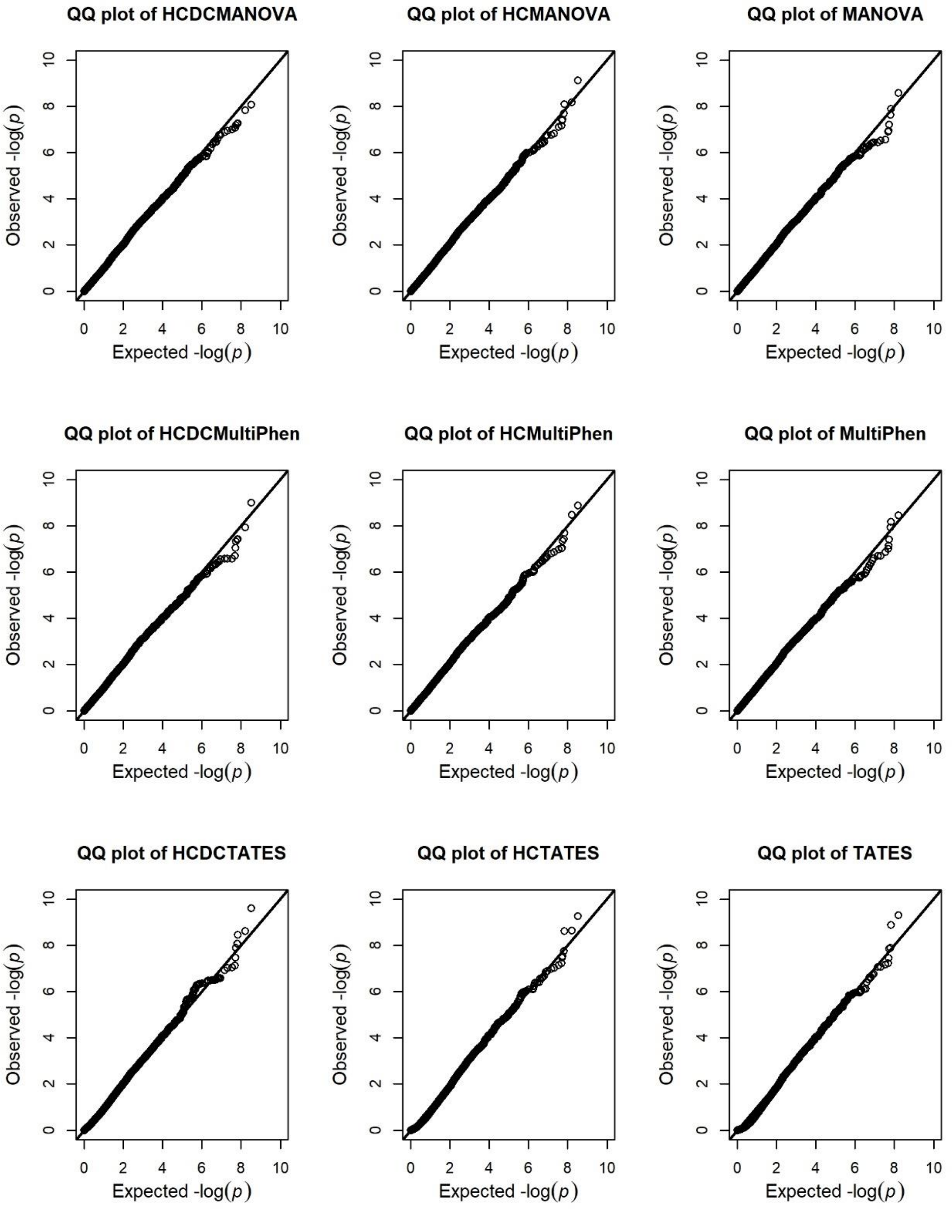


**Supplementary Figure 1. Type I error rate comparisons of the nine methods in Q-Q plot in model 1.** Sample size is $N = 2{,}000$, the number of phenotypes is $M = 16$, $c^2 = 0.5$, $\rho c^2 = 0.1$, and MAF = 0.3. The type I error rate of all the nine methods is estimated using 10,000 replicated samples.

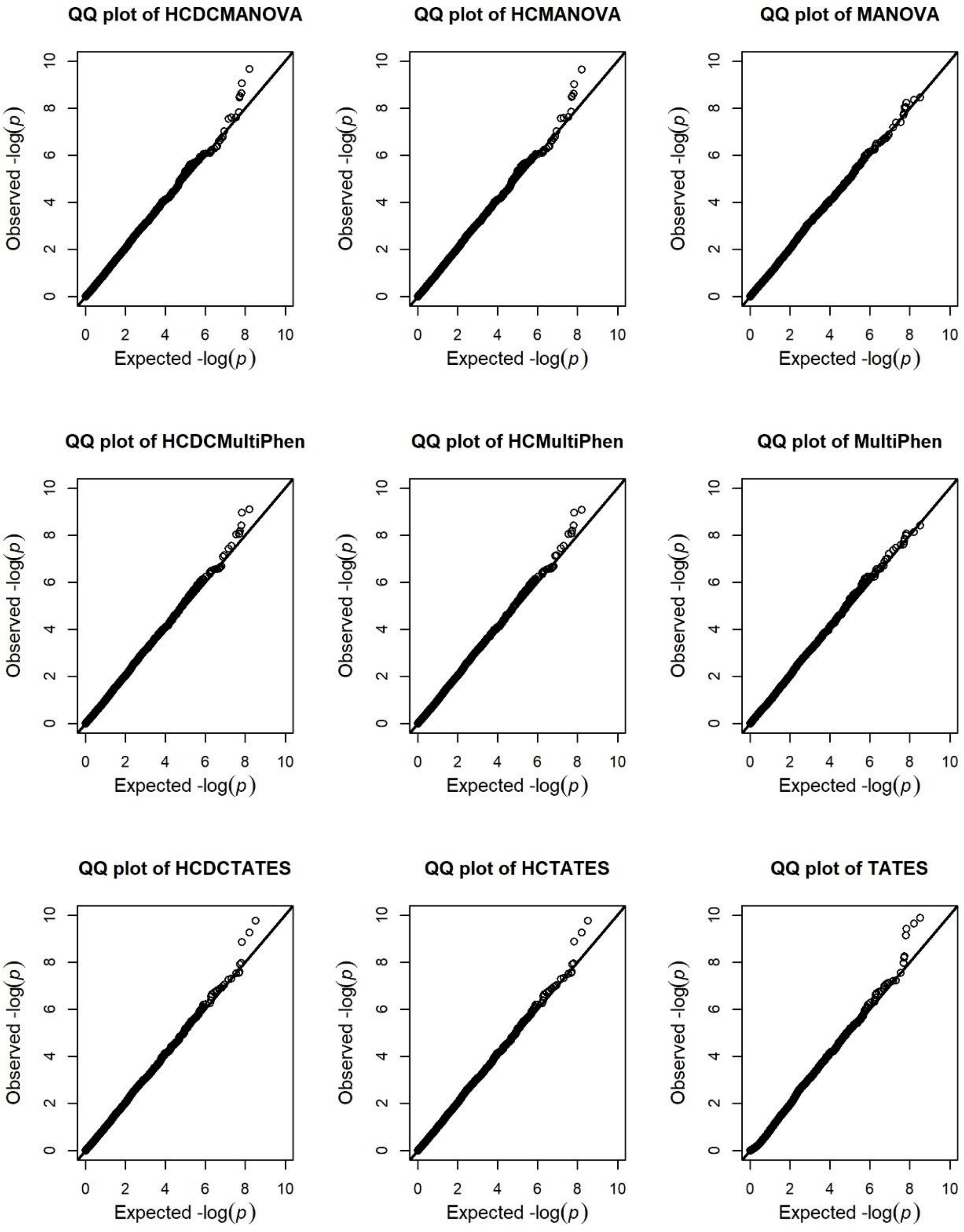


**Supplementary Figure 2. Type I error rate comparisons of the nine methods in Q-Q plot in model 2.** Sample size is $N = 2{,}000$, the number of phenotypes is $M = 16$, $c^2 = 0.5$, $\rho c^2 = 0.1$, and MAF = 0.3. The type I error rate of all the nine methods is estimated using 10,000 replicated samples.

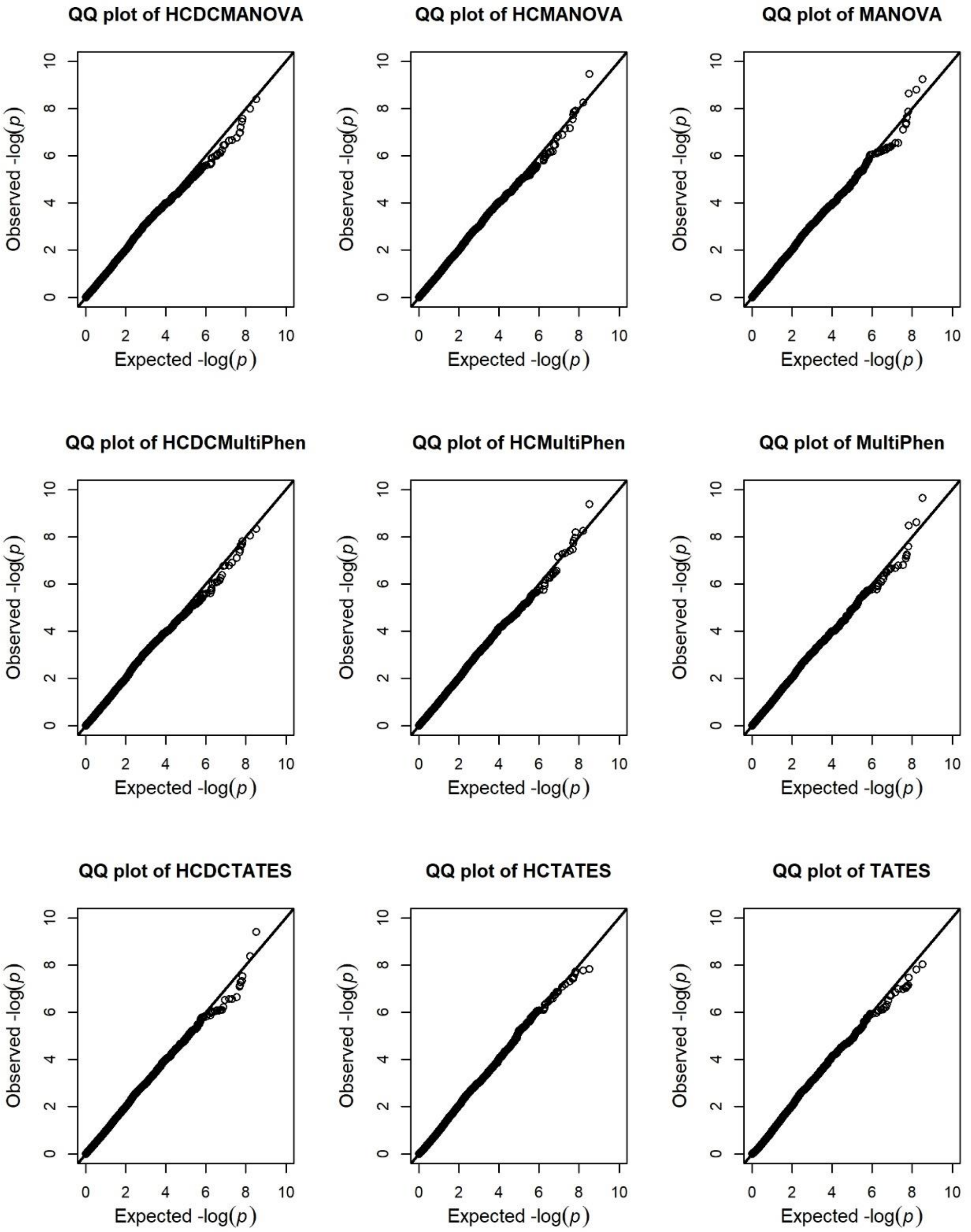


**Supplementary Figure 3. Type I error rate comparisons of the nine methods in Q-Q plot in model 3.** Sample size is $N = 2{,}000$, the number of phenotypes is $M = 16$, $c^2 = 0.5$, $\rho c^2 = 0.1$, and MAF = 0.3. The type I error rate of all the nine methods is estimated using 10,000 replicated samples.

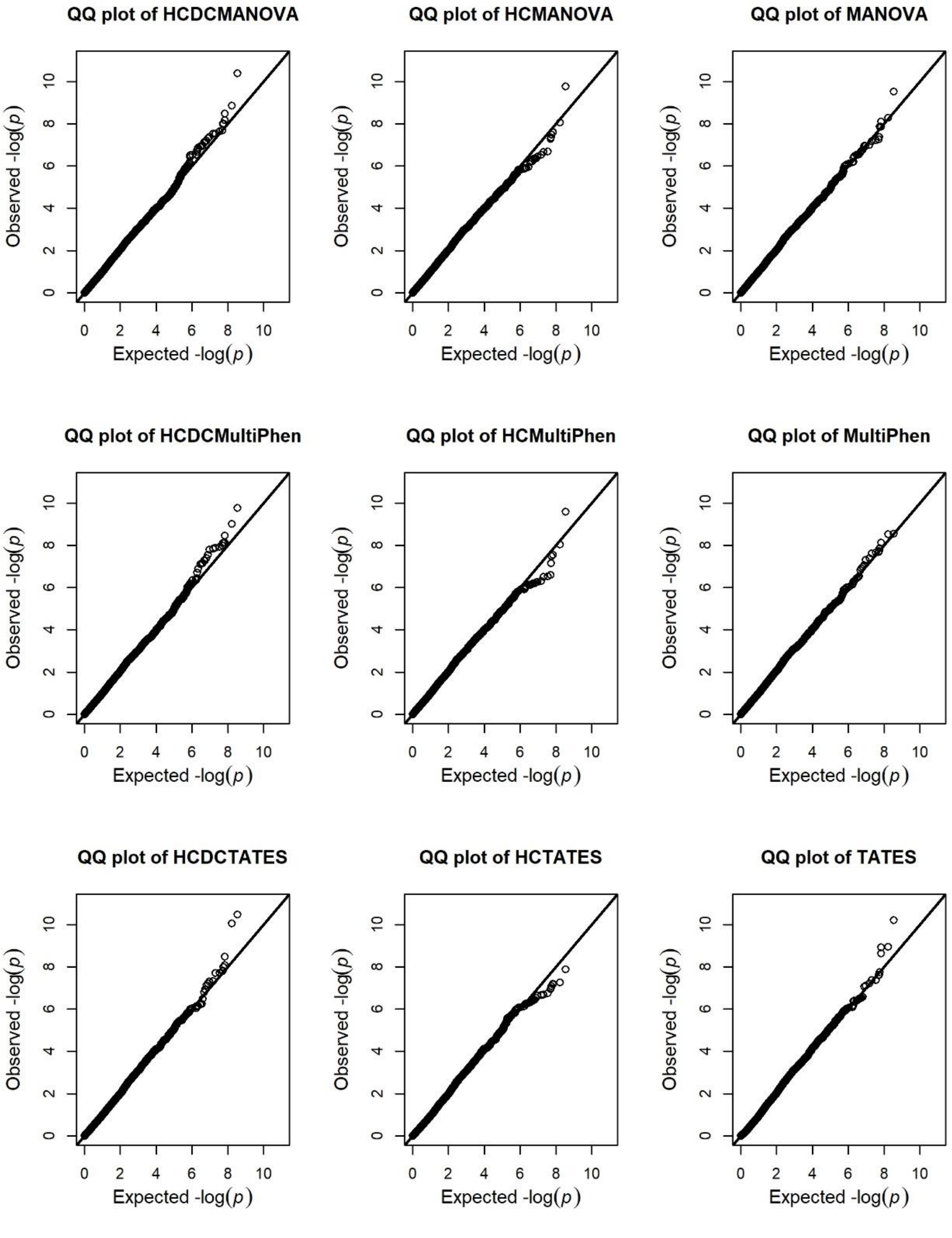


**Supplementary Figure 4. Type I error rate comparisons of the nine methods in Q-Q plot in model 4.** Sample size is $N = 2{,}000$, the number of phenotypes is $M = 16$, $c^2 = 0.5$, $\rho c^2 = 0.1$, and MAF = 0.3. The type I error rate of all the nine methods is estimated using 10,000 replicated samples.

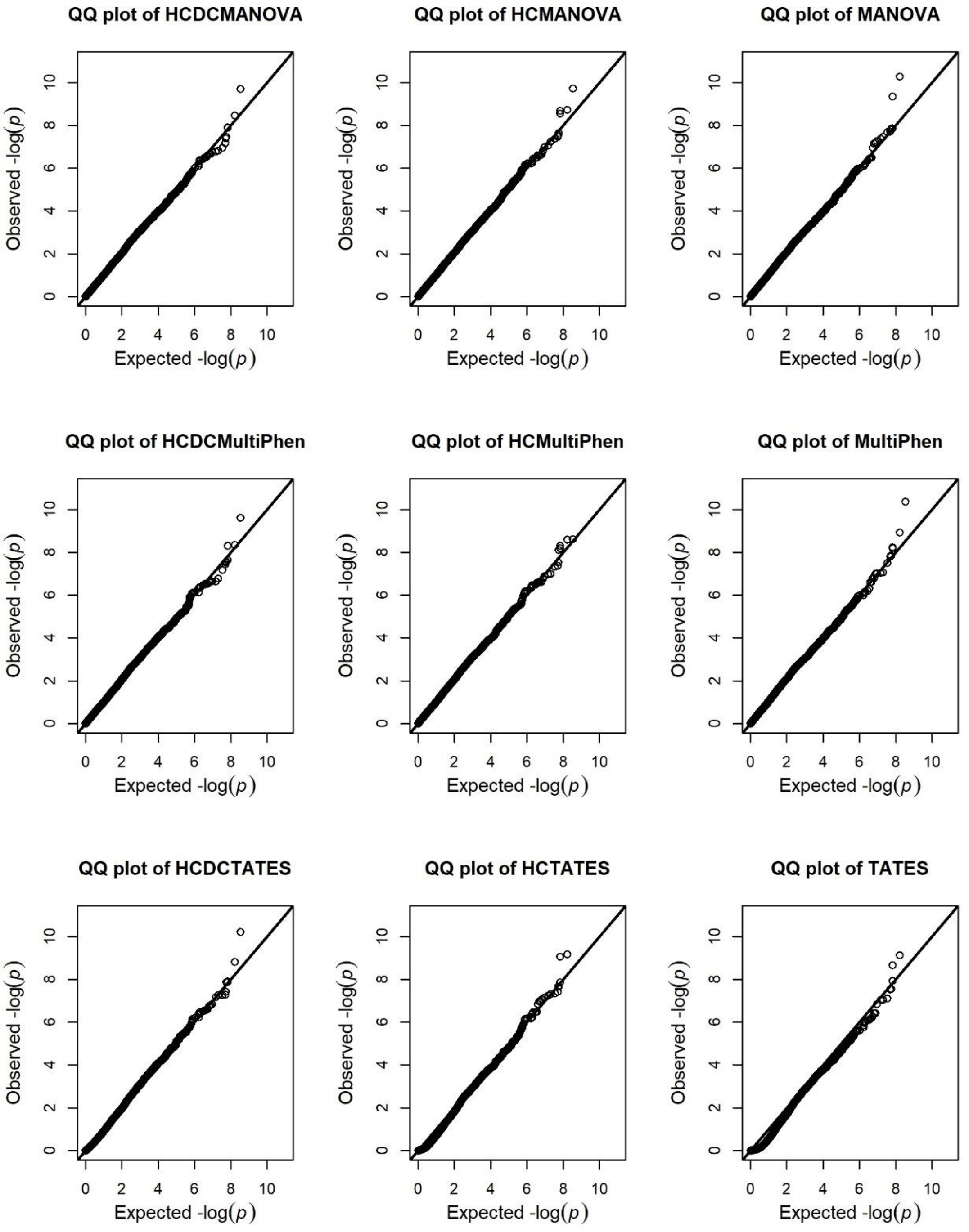


**Supplementary Figure 5. Type I error rate comparisons of the nine methods in Q-Q plot in model 1.** Sample size is $N = 2{,}000$, the number of phenotypes is $M = 32$, $c^2 = 0.5$, $\rho c^2 = 0.1$, and MAF = 0.3. The type I error rate of all the nine methods is estimated using 10,000 replicated samples.

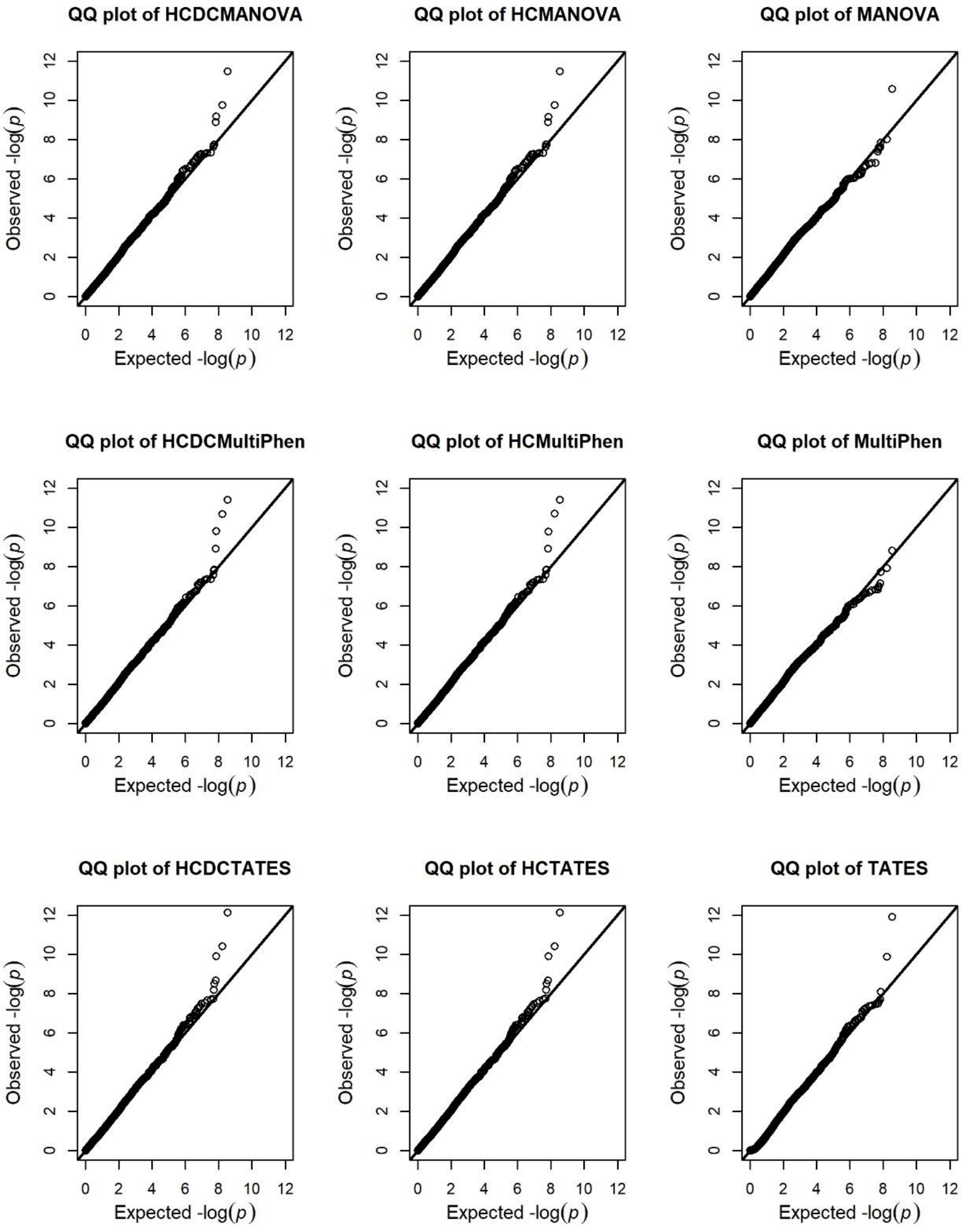


**Supplementary Figure 6. Type I error rate comparisons of the nine methods in Q-Q plot in model 2.** Sample size is $N = 2{,}000$, the number of phenotypes is $M = 32$, $c^2 = 0.5$, $\rho c^2 = 0.1$, and MAF = 0.3. The type I error rate of all the nine methods is estimated using 10,000 replicated samples.

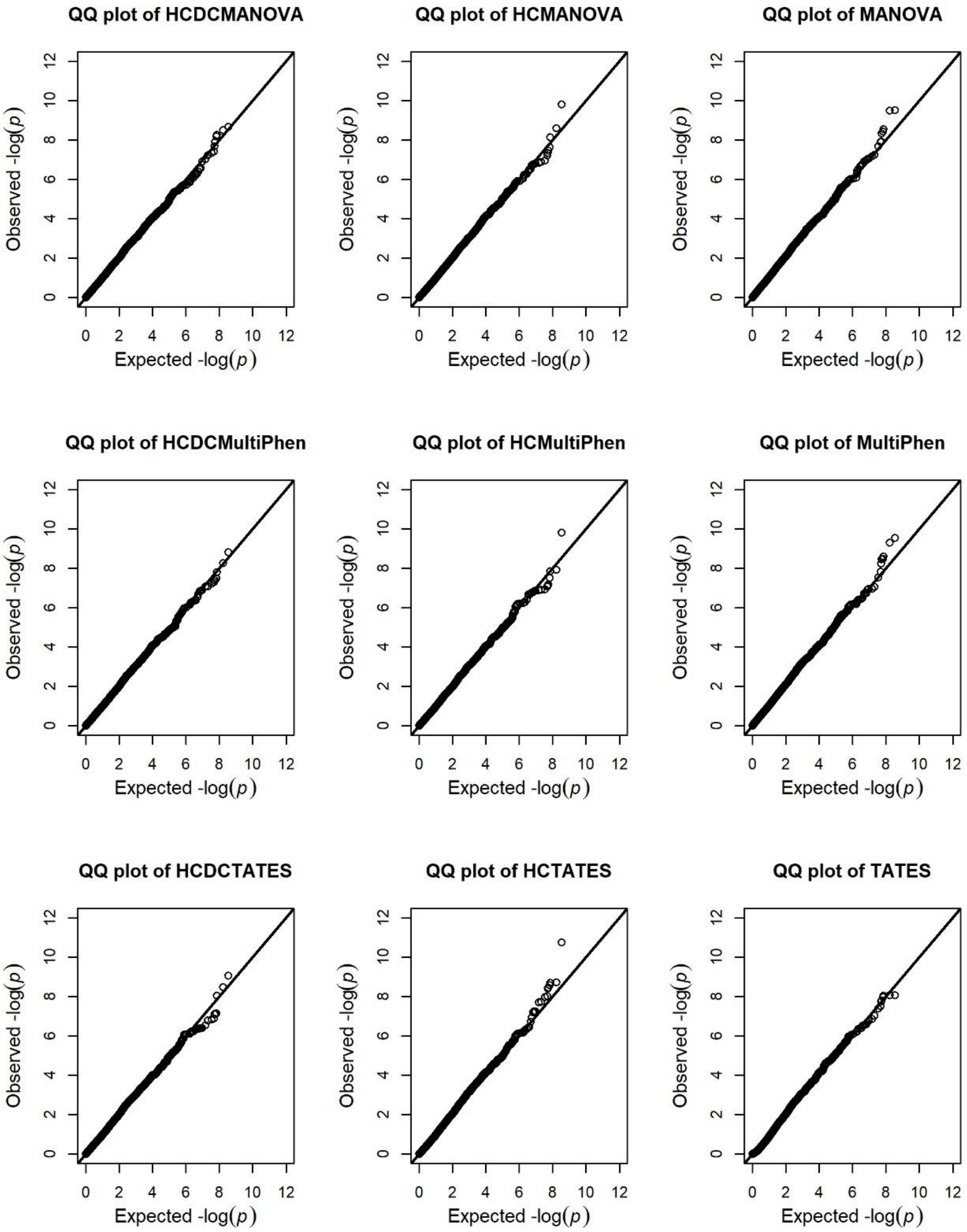


**Supplementary Figure 7. Type I error rate comparisons of the nine methods in Q-Q plot in model 3.** Sample size is $N = 2{,}000$, the number of phenotypes is $M = 32$, $c^2 = 0.5$, $\rho c^2 = 0.1$, and MAF = 0.3. The type I error rate of all the nine methods is estimated using 10,000 replicated samples.

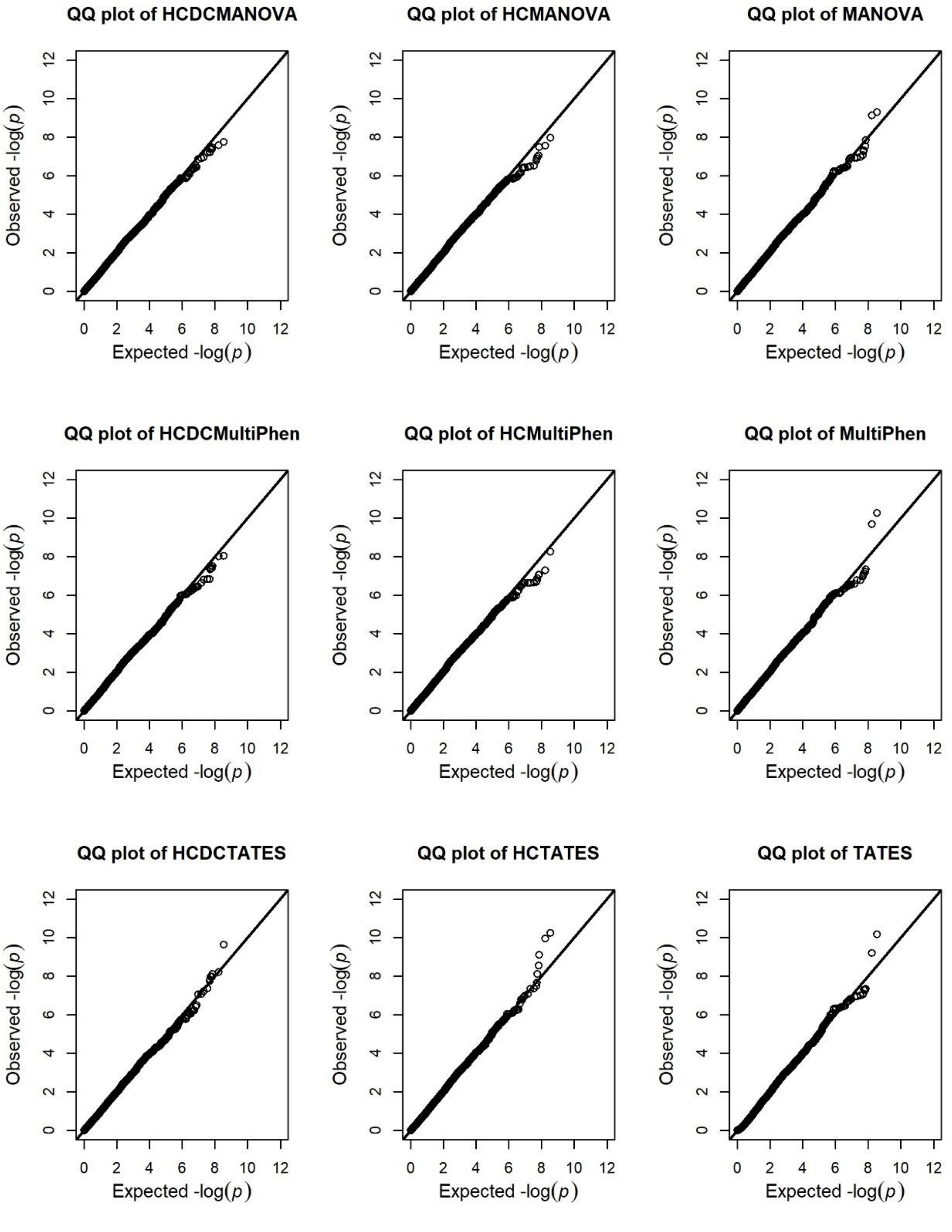


**Supplementary Figure 8. Type I error rate comparisons of the nine methods in Q-Q plot in model 4.** Sample size is $N = 2{,}000$, the number of phenotypes is $M = 32$, $c^2 = 0.5$, $\rho c^2 = 0.1$, and MAF = 0.3. The type I error rate of all the nine methods is estimated using 10,000 replicated samples.

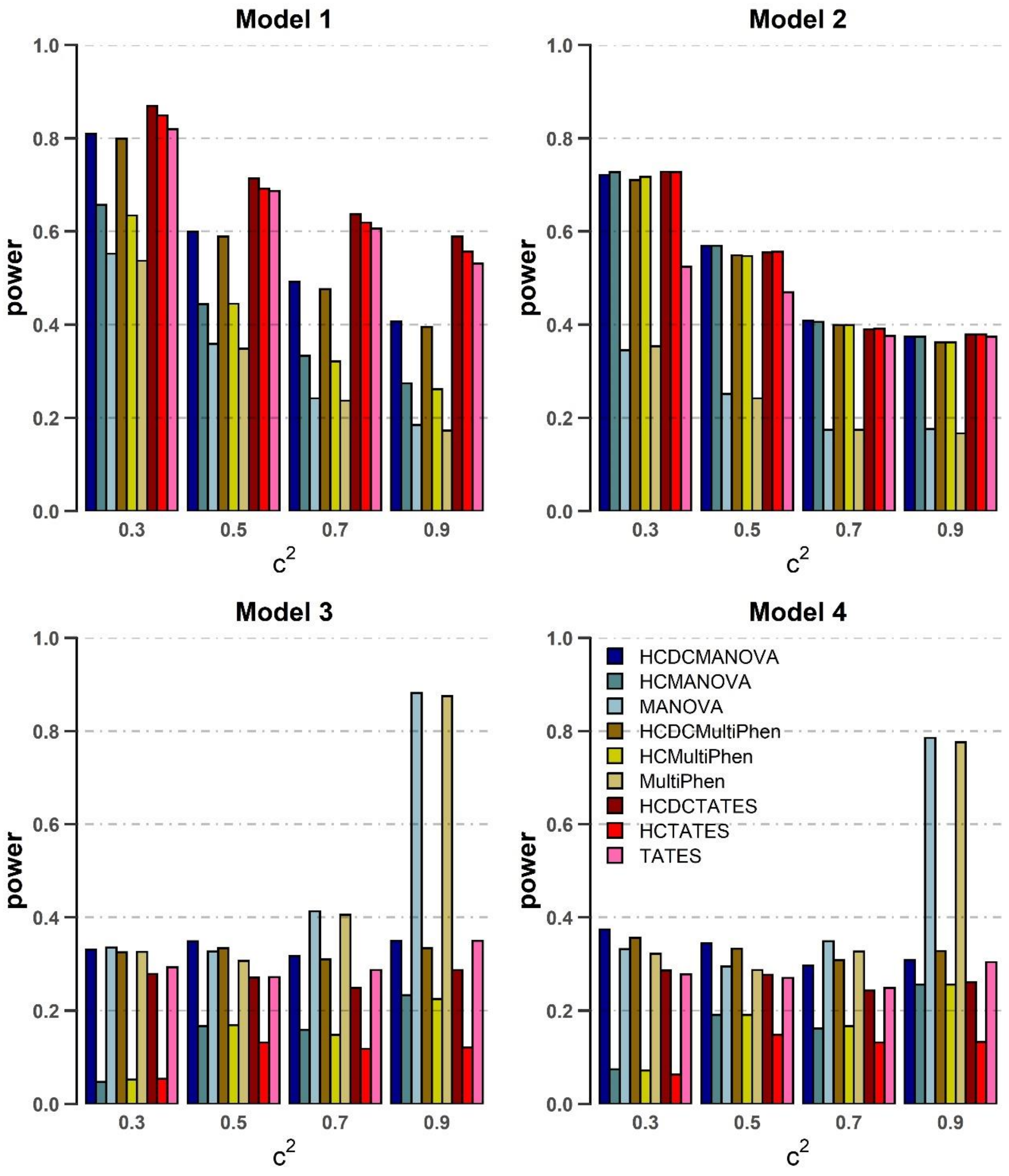


**Supplementary Figure 9. Power comparisons of the nine methods as a function of $c^2$ in the four models.** Sample size $N = 2{,}000$, the number of phenotypes $M = 16$, $c^2 = 0.5$, $\rho c^2 = 0.1$, and MAF = 0.3. $\beta = 0.07$ for model 1; $\beta = 0.06$ for model 2; $\beta = 0.03$ for model 3; $\beta = 0.0275$ for model 4. The power of all the nine methods is evaluated by 1,000 replicated samples at a significance level of 0.05.

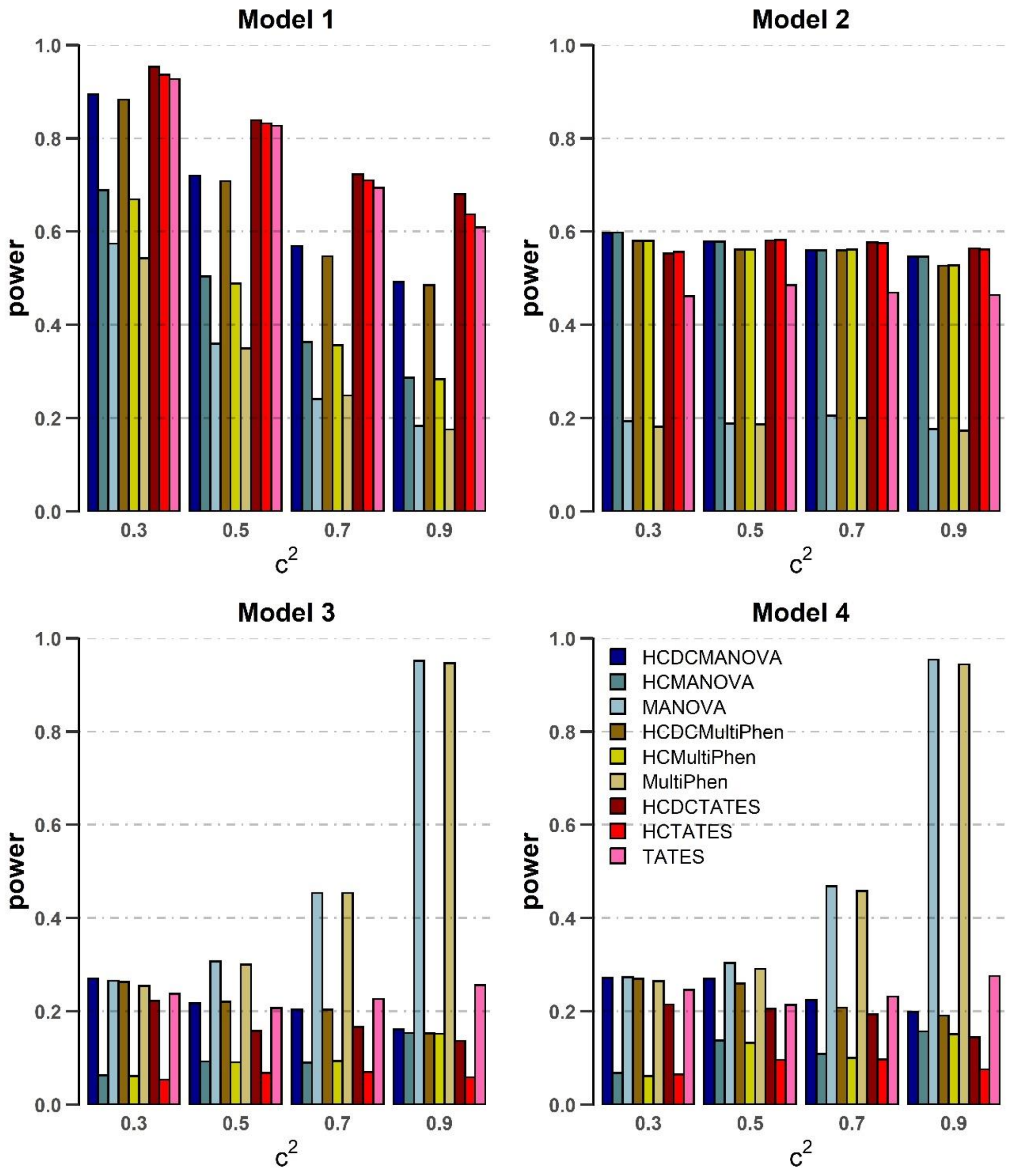


**Supplementary Figure 10. Power comparisons of the nine methods as a function of $c^2$ in the four models.** Sample size $N = 2{,}000$, the number of phenotypes $M = 32$, $c^2 = 0.5$, $\rho c^2 = 0.1$, and MAF = 0.3. $\beta = 0.08$ for model 1; $\beta = 0.06$ for model 2; $\beta = 0.015$ for models 3 and 4. The power of all the nine methods is evaluated by 1,000 replicated samples at a significance level of 0.05.

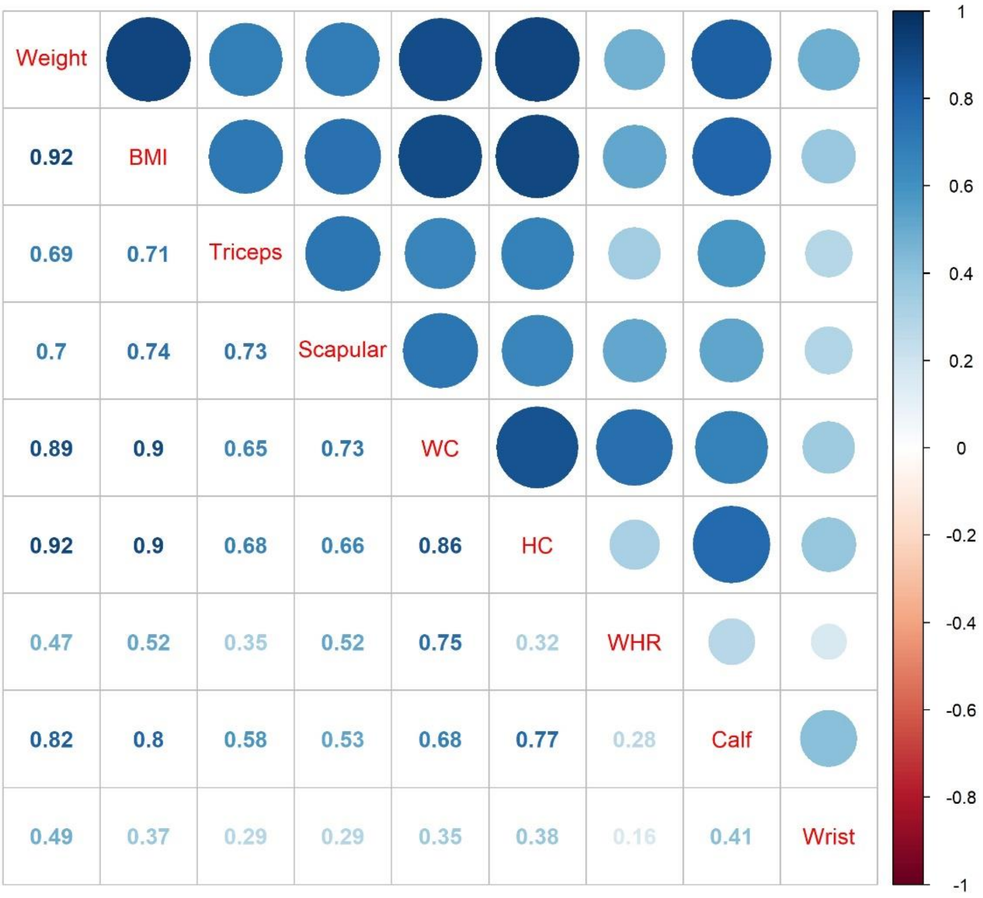


**Supplementary Figure 11. Correlation coefficient matrix for obesity indicators in ARIC.** BMI is body mass index; Triceps is average skinfold thickness of triceps brachii; Scapular is mean subscapular skinfold thickness; WC is waist; HC is hip girth; WHR is waist to hip ratio; Calf is calf girth; and Wrist is wrist breadth.

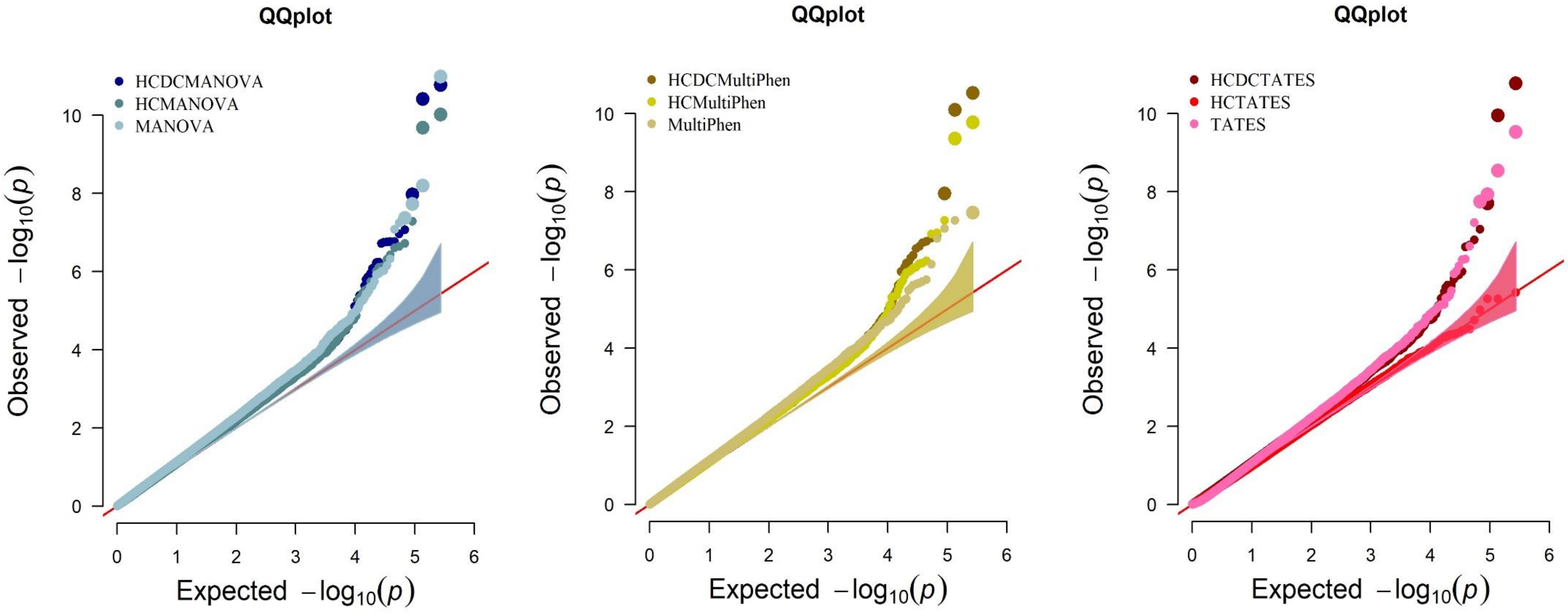


**Supplementary Figure 12. The Q-Q plots after employing HCDCMANOVA, HCMANOVA, MANOVA, HCDCMultiPhen, HCMultiPhen, MultiPhen, HCDCTATES, HCTATES, and TATES in ARIC.** The p-values in vertical axis denote the p-values –log transformed after using distinct methods. The horizontal axis represents the -log transformed value of the standard uniform distribution. The shaded area represents the 95% confidence interval for the standard -log10(*p*) value. The larger dots in the upper right corner of each plot denote SNPs with significance threshold of $5\times10^{-8}$.

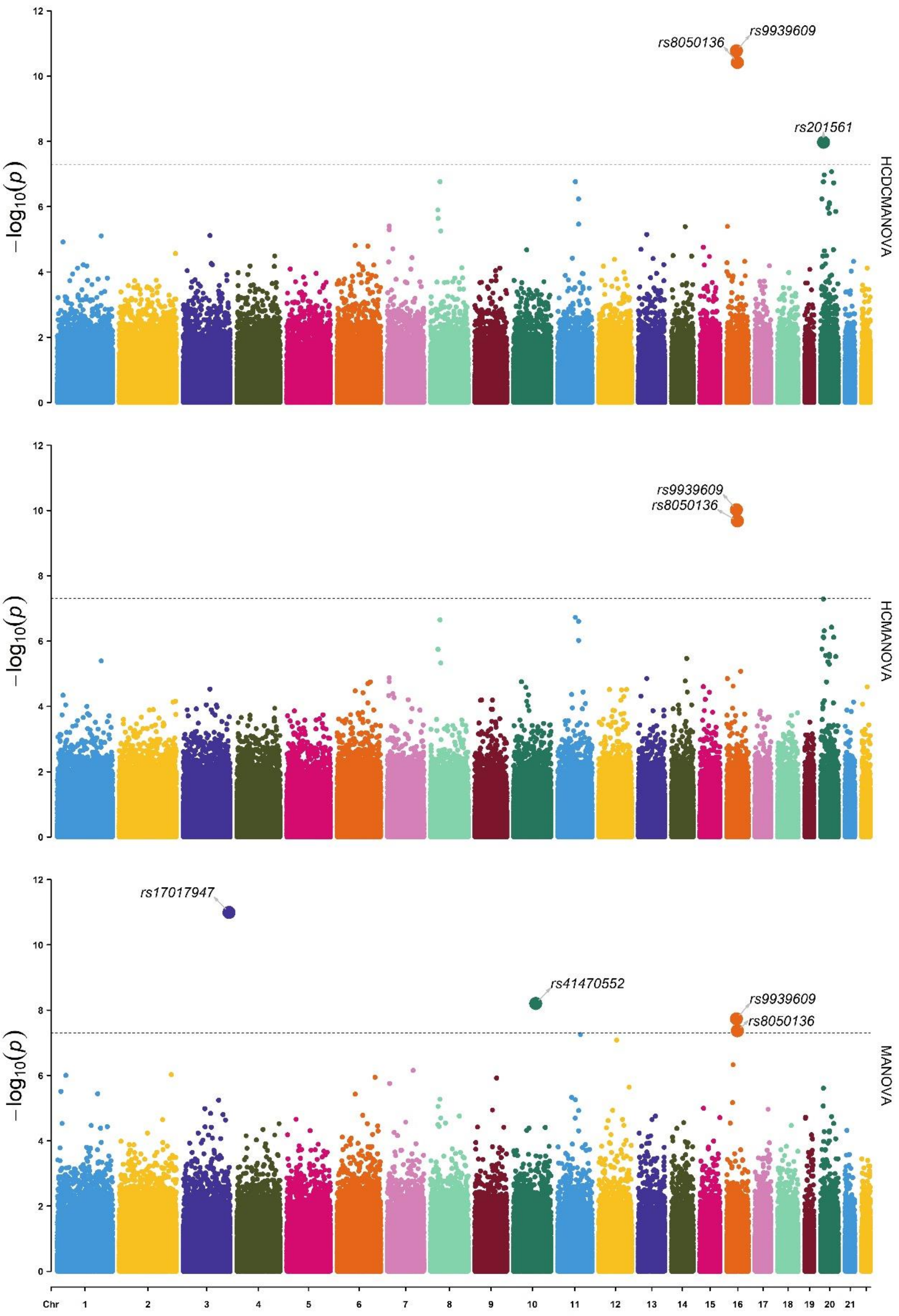


**Supplementary Figure 13. The Manhattan plots after employing HCDCMANOVA, HCMANOVA, and MANOVA in ARIC.** The p-values in vertical axis denote the p-values –log transformed after using distinct methods. The horizontal axis represents the chromosome. The larger dots in the upper of each plot denote SNPs with significance threshold of $5\times10^{-8}$.

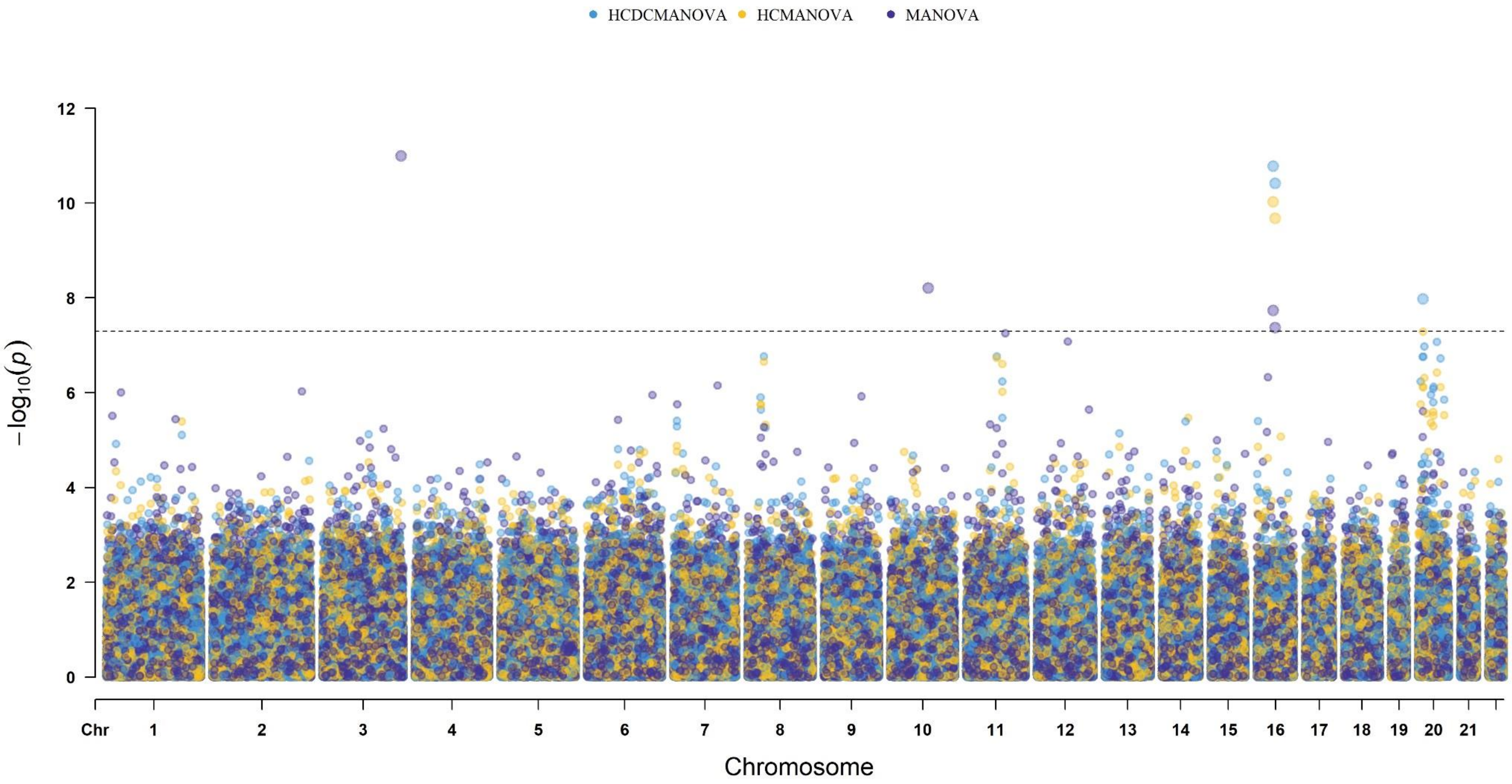


**Supplementary Figure 14. The merged Manhattan plot after employing HCDCMANOVA, HCMANOVA, and MANOVA in ARIC.** The p-values in vertical axis denote the p-values –log transformed after using distinct methods. The horizontal axis represents the chromosome. The larger dots in the upper of each plot denote SNPs with significance threshold of $5\times10^{-8}$.

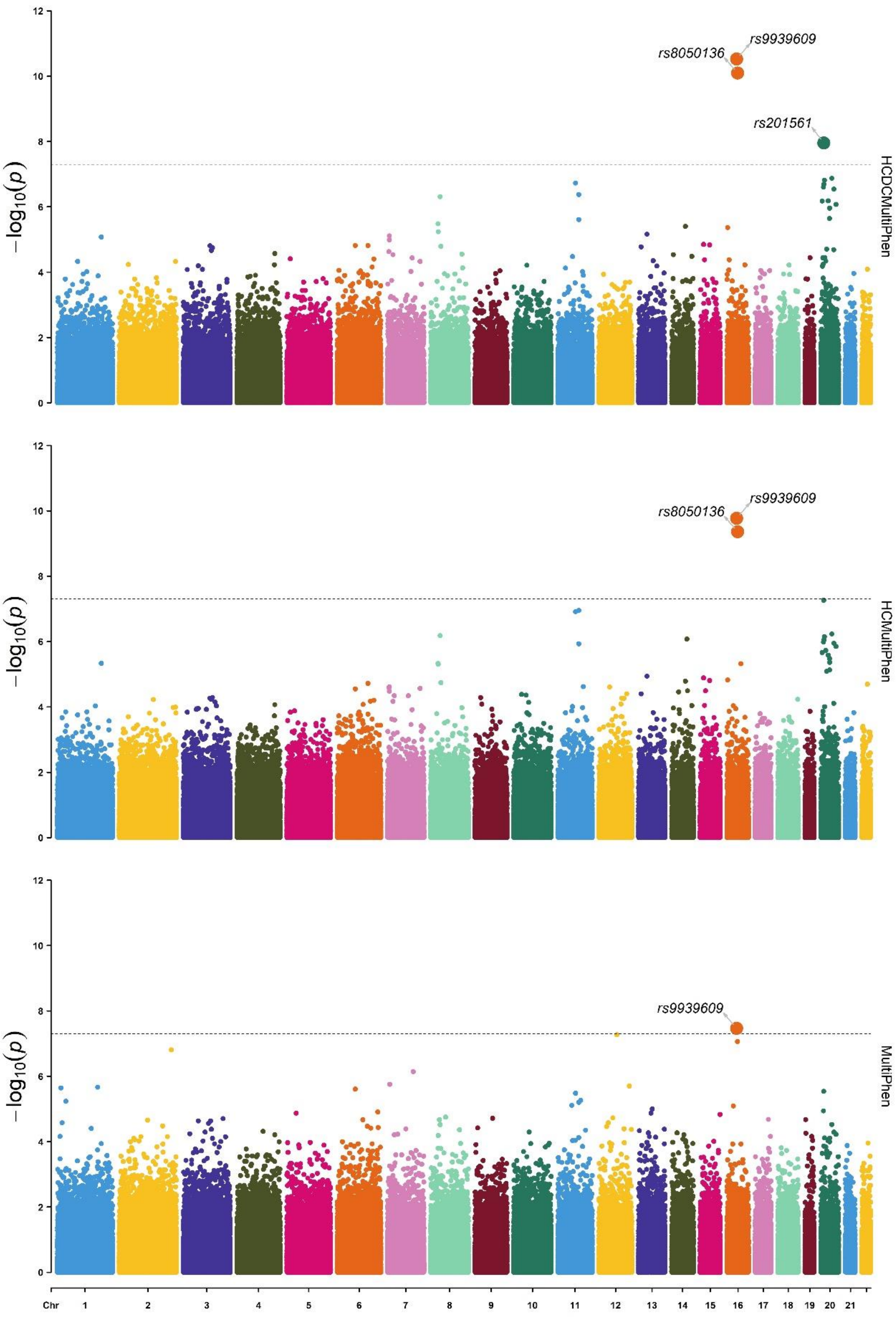


**Supplementary Figure 15. The Manhattan plots after employing HCDCMultiPhen, HCMultiPhen, and MultiPhen in ARIC.** The p-values in vertical axis denote the p-values –log transformed after using distinct methods. The horizontal axis represents the chromosome. The larger dots in the upper of each plot denote SNPs with significance threshold of $5\times10^{-8}$.

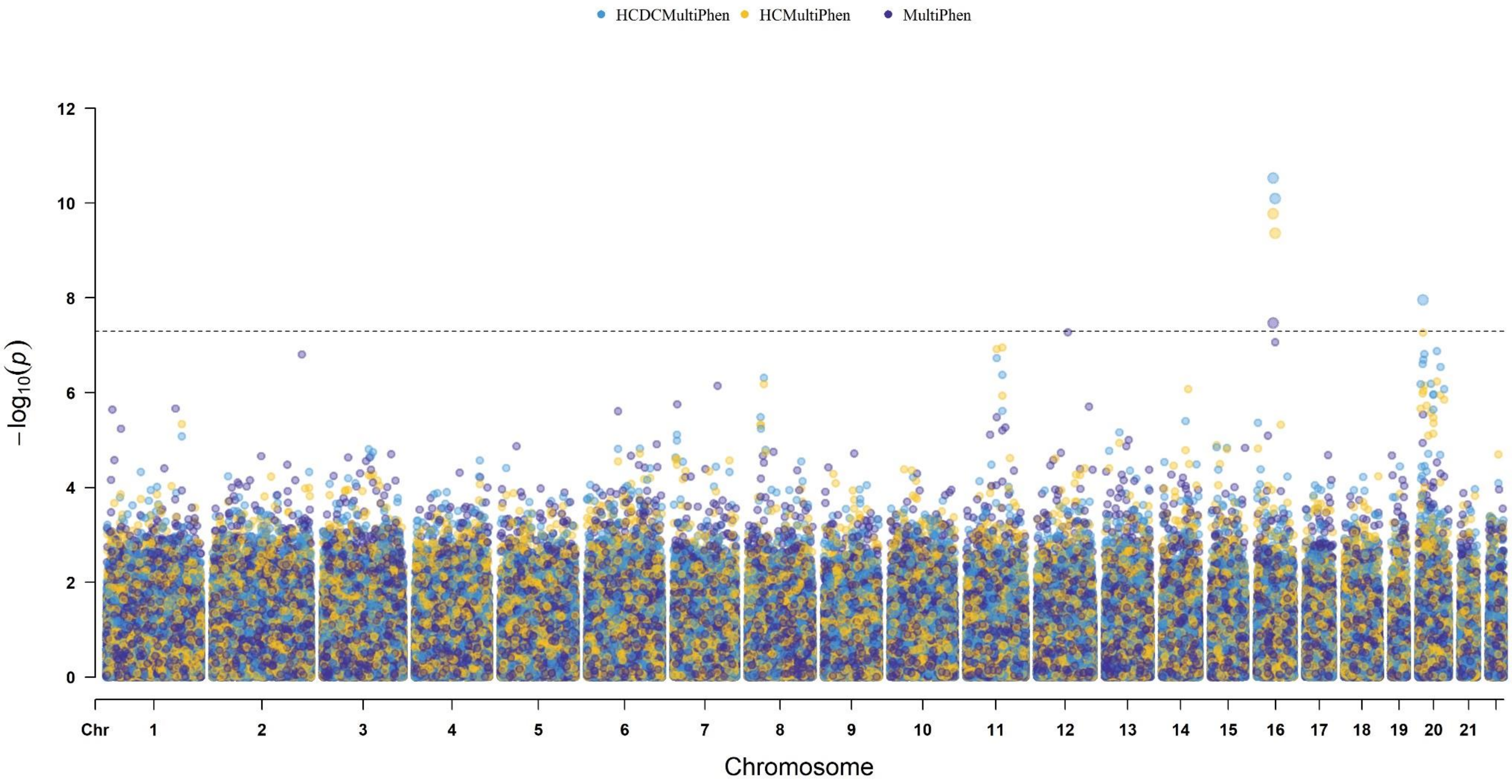


**Supplementary Figure 16. The merged Manhattan plot after employing HCDCMultiPhen, HCMultiPhen, and MultiPhen in ARIC.** The p-values in vertical axis denote the p-values –log transformed after using distinct methods. The horizontal axis represents the chromosome. The larger dots in the upper of each plot denote SNPs with significance threshold of $5\times10^{-8}$.

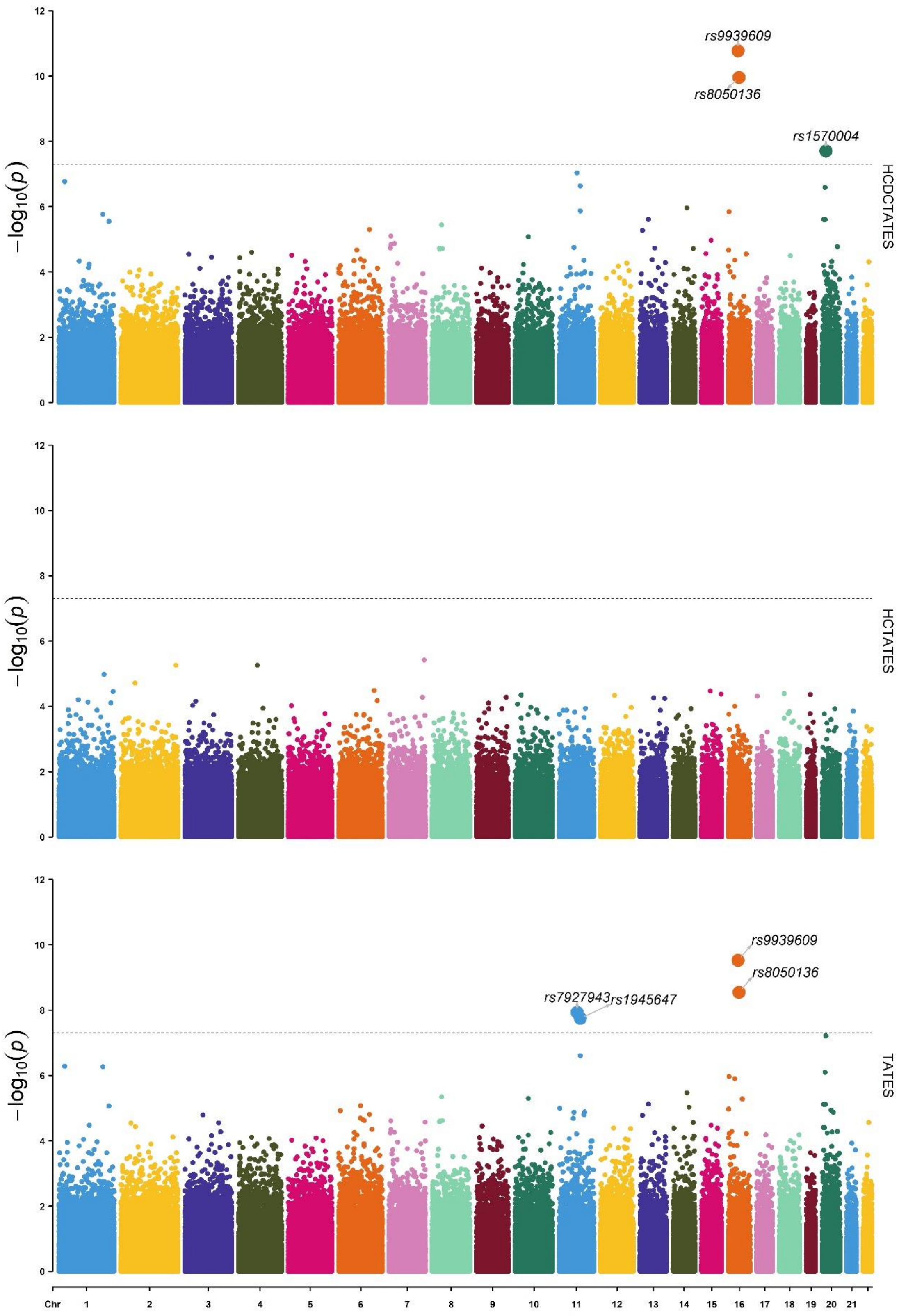


**Supplementary Figure 17. The Manhattan plots after employing HCDCTATES, HCTATES, and TATES in ARIC.** The p-values in vertical axis denote the p-values –log transformed after using distinct methods. The horizontal axis represents the chromosome. The larger dots in the upper of each plot denote SNPs with significance threshold of $5\times10^{-8}$.

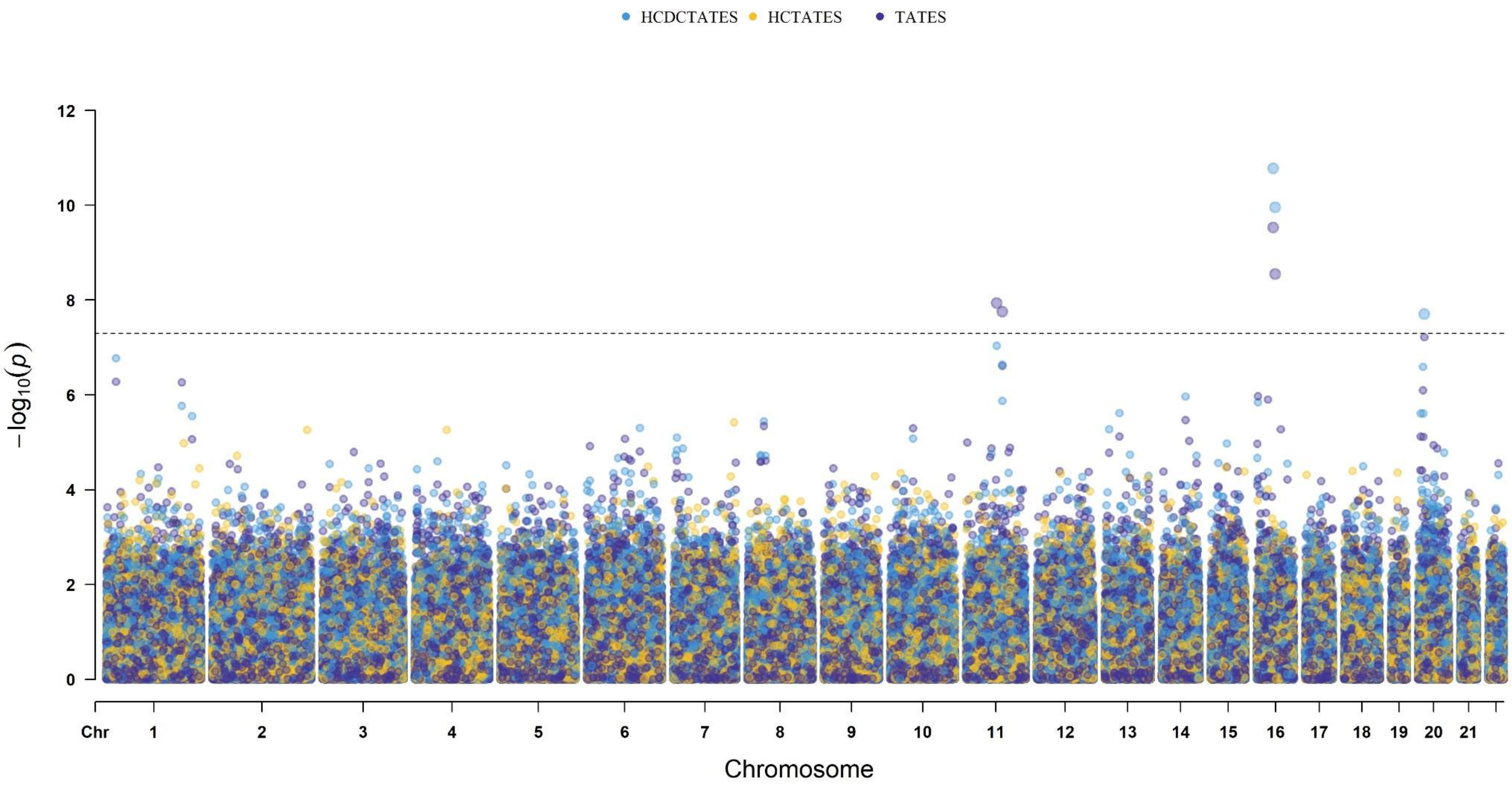


**Supplementary Figure 18. The merged Manhattan plot after employing HCDCTATES, HCTATES, and TATES in ARIC.** The p-values in vertical axis denote the p-values –log transformed after using distinct methods. The horizontal axis represents the chromosome. The larger dots in the upper of each plot denote SNPs with significance threshold of $5\times10^{-8}$.

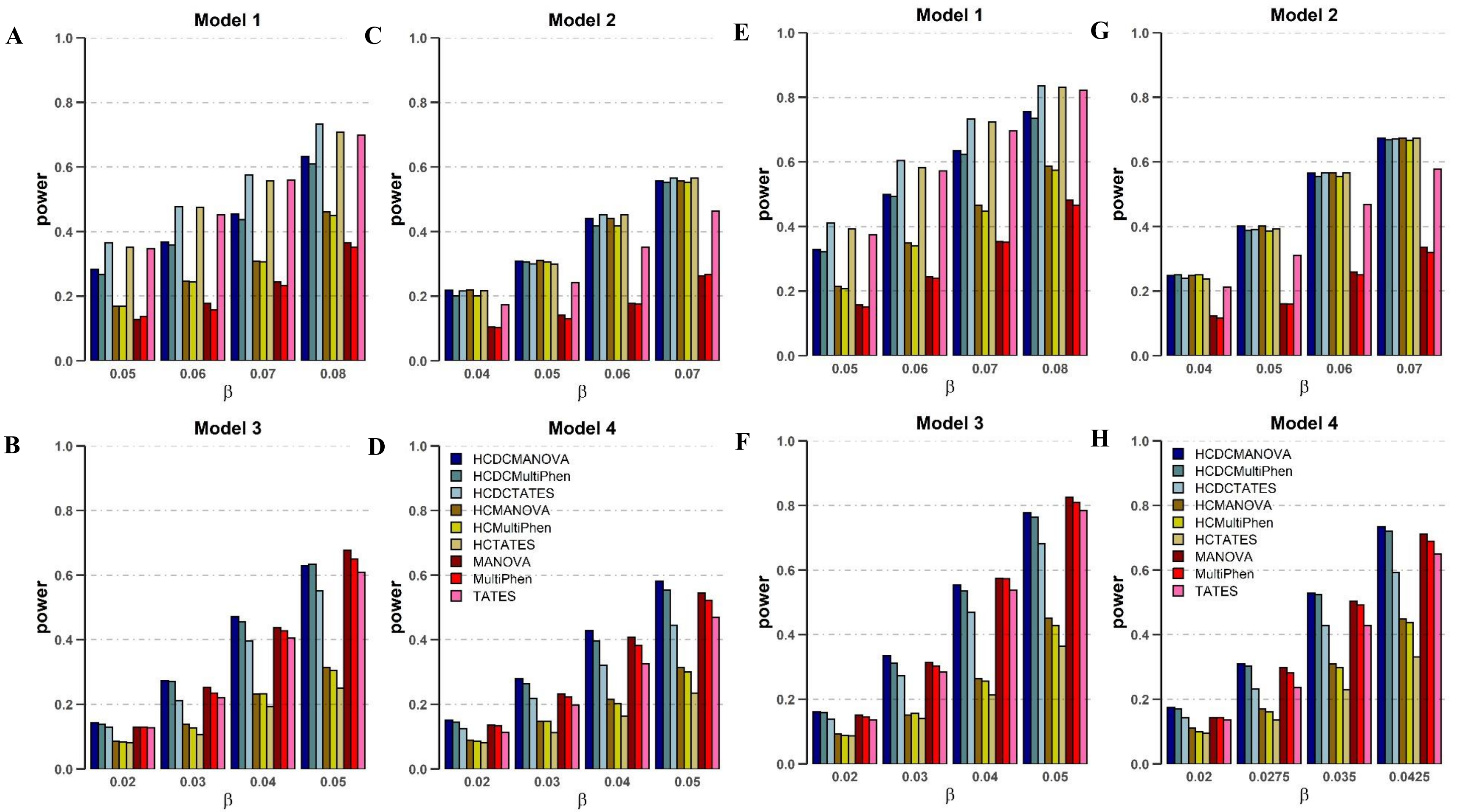


**Supplementary Figure 19. Power comparisons of the nine methods as a function of β in the different MAF.** Sample size $N = 2{,}000$, the number of phenotypes $M = 16$, $c^2 = 0.5$, $\rho c^2 = 0.1$, MAF = 0.2 (figures A, B, C and D) and MAF = 0.3 (figures E, F, G and H). The power of all the nine methods is evaluated by 1,000 replicated samples at a significance level of 0.05.

**Supplementary Table 1.** The clustering results of HCDC for the setting of sample size $N = 2{,}000$, the number of phenotypes $M = 16$, $c^2 = 0.5$, $pc^2 = 0.1$, MAF = 0.2 and MAF = 0.3.

| | **Clustering results of HCDC under MAF=0.2** | **Clustering results of HCDC under MAF=0.3** |
|---|---|---|
| **Model 1** | The number of clusters are not constant, and the majority of simulations contain one large group including various phenotypes, the other groups contain only one phenotype. | Same with MAF=0.2 in Model 1. |
| **Model 2** | The number of clusters are constant. Two clusters are observed. One of the cluster contains phenotypes 1-8, and the other contains phenotypes 9-16, which are corresponded to the within factor phenotypes. | Same with MAF=0.2 in Model 2. |
| **Model 3** | The number of clusters are constant. four clusters are observed. One of the cluster contains phenotypes 1-4, the second cluster contains phenotypes 4-8, the third cluster contains phenotypes 9-12, and the last cluster contains phenotypes 13-16, which are corresponded to the within factor phenotypes. | Same with MAF=0.2 in Model 3 |
| **Model 4** | The number of clusters are constant. four clusters are observed. One of the cluster contains phenotypes 1-4, the second cluster contains phenotypes 4-8, the third cluster contains phenotypes 9-12, and the last cluster contains phenotypes 13-16, which are corresponded to the within factor phenotypes. | Same with MAF=0.2 in Model 4. |

**Supplementary Table 2.** Display of significant SNPs and the corresponding *p*-values in the analysis of ARIC via single phenotype association test and randomly pick *K* phenotypes and apply MANOVA/MultiPhen/TATES on them where *K* is the number of clusters from HCDC.

| Chr | SNP | Weight | BMI | Triceps | Scapular | WC | HC | WHtR | Calf | Wrist | MANOVA | TATES | Multiphe |
|---|---|---|---|---|---|---|---|---|---|---|---|---|---|
| 11 | rs7927943 | 4.71E-07 | 7.06E-08 | 1.09E-06 | 2.26E-05 | 1.17E-07 | 1.64E-09 | 0.029 | 2.99E-05 | 0.215 | 3.07E-06 | 7.82E-07 | 3.47E-06 |
| 11 | rs1945647 | 6.37E-07 | 1.80E-07 | 3.36E-06 | 1.25E-05 | 8.59E-07 | 3.08E-09 | 0.099 | 3.30E-05 | 0.185 | 4.09E-06 | 9.14E-07 | 2.92E-06 |
| 16 | rs9939609 | 4.92E-10 | 5.38E-11 | 1.96E-07 | 3.38E-09 | 4.45E-10 | 2.40E-08 | 7.79E-06 | 1.54E-09 | 0.264 | 1.01E-09 | 6.77E-10 | 1.48E-09 |
| 16 | rs8050136 | 2.14E-09 | 5.48E-10 | 5.57E-07 | 1.46E-08 | 1.44E-09 | 1.16E-07 | 8.60E-06 | 1.86E-08 | 0.602 | 7.49E-09 | 2.81E-09 | 1.21E-08 |

The results show the significant *p*-values and the corresponding SNPs retained for at least one phenotype identified by single phenotype association test or randomly pick *K* phenotypes and apply MANOVA/MultiPhen/TATES on them where *K* is the number of clusters from HCDC. For single phenotype association, the *p*-values less than 5E-09 are considered significant because of the Bonferroni adjustment (5E-08/9). For randomly pick *K* phenotypes and apply MANOVA/MultiPhen/TATES on them, the *p*-values less than 5E-08 are considered significant. SNP, single-nucleotide polymorphism. ARIC, Atherosclerosis Risk in Communities. BMI is body mass index; Triceps is average skinfold thickness of triceps brachii; Scapular is mean subscapular skinfold thickness; WC is waist; HC is hip girth; WHtR is waist to hip ratio; Calf is calf girth; and Wrist is wrist breadth.
